# Real-space measurement of orbital electron populations for Li_1-*x*_CoO_2_

**DOI:** 10.1038/s41467-022-33595-0

**Published:** 2022-10-03

**Authors:** Tongtong Shang, Dongdong Xiao, Fanqi Meng, Xiaohui Rong, Ang Gao, Ting Lin, Zhexin Tang, Xiaozhi Liu, Xinyan Li, Qinghua Zhang, Yuren Wen, Ruijuan Xiao, Xuefeng Wang, Dong Su, Yong-Sheng Hu, Hong Li, Qian Yu, Ze Zhang, Vaclav Petricek, Lijun Wu, Lin Gu, Jian-Min Zuo, Yimei Zhu, Ce-Wen Nan, Jing Zhu

**Affiliations:** 1 Beijing National Laboratory for Condensed Matter Physics, Institute of Physics, Chinese Academy of Sciences, Beijing, 100190 P. R. China; 2 School of Physical Sciences, University of Chinese Academy of Sciences, Beijing, 100049 China; 3 Songshan Lake Materials Laboratory, Dongguan, 523808 P. R. China; 4 State Key Lab of New Ceramics and Fine Processing, School of Materials Science and Engineering, Tsinghua University, Beijing, 100084 P. R. China; 5 School of Materials Science and Engineering, University of Science and Technology Beijing, Beijing, 100083 P. R. China; 6 Department of Materials Science and Engineering, Center of Electron Microscopy and State Key Laboratory of Silicon Materials, Zhejiang University, Hangzhou, 310027 P. R. China; 7 Institute of Physics, Academy of Sciences of the Czech Republic, Praha, 180 40 Czech Republic; 8 Condensed Matter Physics and Materials Science Division, Brookhaven National Laboratory, Upton, New York 11973 USA; 9 Department of Materials Science and Engineering, University of Illinois at Urbana Champaign, 1304 W Green St, Urbana, 61801 USA; 10 Beijing National Center for Electron Microscopy, Laboratory of Advanced Materials, Department of Materials Science and Engineering, Tsinghua University, Beijing, 100084 P. R. China

**Keywords:** Imaging techniques, Electronic properties and materials

## Abstract

The operation of lithium-ion batteries involves electron removal from and filling into the redox orbitals of cathode materials, experimentally probing the orbital electron population thus is highly desirable to resolve the redox processes and charge compensation mechanism. Here, we combine quantitative convergent-beam electron diffraction with high-energy synchrotron powder X-ray diffraction to quantify the orbital populations of Co and O in the archetypal cathode material LiCoO_2_. The results indicate that removing Li ions from LiCoO_2_ decreases Co *t*_*2g*_ orbital population, and the intensified covalency of Co–O bond upon delithiation enables charge transfer from O *2p* orbital to Co *e*_*g*_ orbital, leading to increased Co *e*_*g*_ orbital population and oxygen oxidation. Theoretical calculations verify these experimental findings, which not only provide an intuitive picture of the redox reaction process in real space, but also offer a guidance for designing high-capacity electrodes by mediating the covalency of the TM–O interactions.

## Abstract (web summary)

Experimentally probing the orbital population is highly desirable to resolve the redox mechanism of cathodes materials. Here the authors quantify the orbital populations of Co and O in LiCoO_2_ and identify the ligand-to-metal charge transfer.

## Introduction

The orbital population of valence electrons is an important determinant of the physical and chemical properties of functional materials because a large variety of functional properties is controlled by the delicate sensitivity of charge transfer and the orbital electron occupations^1, 2^. In recent years, probing and manipulating the orbital configurations in transition-metal oxides have been the backbone of intensive research into physical and chemical fields, such as energy storage and conversion^3^, electronic devices^4^, and catalysis^5^. Among these, the ever-increasing energy consumption in modern society has awakened the strong demand for energy storage devices with higher energy density, durability, and safety^6^.

Lithium-ion batteries (LIBs) have transformed portable electronic devices and become the preferred technology to enable the electrification of road transport due to their outstanding performances in comparison with other rechargeable batteries^7^. Despite the fact that great progress has been made in terms of cathodes, anodes, and electrolytes, the capacity of oxide cathode materials is still the primary bottleneck limiting performances of LIBs^8–10^. As such, much attention has been directed toward revealing the fundamental redox mechanism underlying delithiation and lithiation of cathode materials during battery operation so that novel insights can be obtained to guide the optimization and design of cathode materials. In the classic paradigm, it is tacitly assumed that the electrochemical (de)lithiation of conventional oxide cathode materials is accompanied by the extraction/insertion of electrons from/into the *d*-orbitals of the transition-metal (TM) ions, indicating that cationic redox chemistry dominates the electrochemical processes of cathode materials. While it has recently become increasingly clear that the lattice oxygen besides TM ions can also participate in the charge compensation in classical layered oxide cathode materials including LiCoO_2_^11–13^ and LiNi_0.33_Co_0.33_Mn_0.33_O_2_^14, 15^, especially at high charging cut-off voltage to extract more lithium ions. The intriguing oxygen redox has been attributed to the enhanced TM–O hybridization upon charging^13^, this is different from oxygen activity reported in the Li-rich layered cathode materials, in which non-bonding oxygen *2p* electrons has been proposed to explain the origin of capacity at high-voltage plateau (e.g., 4.5 V vs Li/Li^+^)^16^. However, it is worth noting that the identification of redox couples has mainly been realized through spectroscopical methods, such as electron energy loss spectroscopy (EELS), X-ray photoemission spectroscopy (XPS), X-ray absorption spectroscopy (XAS), and X-ray resonant inelastic scattering (RIXS), but it is still difficult to map valence electron distributions and quantify the electron population of redox orbitals or the interplay between TM and O ions.

Since the electron density around atoms in a crystal is coded in the structure factors through Fourier transformation, experimentally probing the valence electron distribution can be achieved by the refinement of the structure factors measured by X-ray diffraction and/or quantitative convergent-beam electron diffraction (QCBED). Specifically, CBED shows better sensitivity at low scattering angles than X-ray diffraction, and the small electron probe makes it more suitable for the study of valence electron and bonding interactions from perfect sample areas^17–19^. According to the many-beam dynamic diffraction theory, the intensity distribution of CBED patterns can be obtained by solving the Schrödinger equation under a periodic potential field in crystals, which can then be compared with experimental patterns to obtain the low-order structure factors^18, 20^. On the other hand, low-temperature, short-wavelength, and high-resolution (sinθ/λ_max_ ≥ 1.0 Å^−1^) synchrotron powder X-ray diffraction (SPXRD) with negligible absorption effects can accurately measure the high-order structure factors and the Debye–Waller factors of small-sized particles. The complementary acquisition of CBED and SPXRD patterns at the same temperature ensures that the Debye–Waller factors are the same, making the merging of experimental results more accurate and reliable than when using either technique alone. Therefore, the combination of QCBED and SPXRD could enable direct visualization of real-space electron density distributions and quantify orbital populations and charge transfer around bonding atoms in cathode materials, thereby definitely resolving the redox orbitals.

In this work, we applied QCBED and high-energy SPXRD to typical layered oxide cathode material LiCoO_2_, which dominates portable devices due to high volumetric energy density, but its reaction mechanism at high voltage still remains elusive^13, 21^. By accurately measuring the structure factors of Li_1-*x*_CoO_2_ (*x* = 0, 0.4, 0.6, 0.7), we successfully mapped the valence electron density of LiCoO_2_ with different states of charge (SOCs) through the multipole refinement of structure factors. In addition, the corresponding *3d*-orbital populations of Co are also quantified based on the relationship between the multipole population parameters and *d*-orbital occupancies of transition-metal atoms. Along with theoretical calculations, we found that the extraction of Li ions from the LiCoO_2_ cathode not only leads to the loss of electrons from the Co *t*_*2g*_ orbital, but also causes an electron gain in the Co *e*_*g*_ orbital and an increased hole density in the O *2p* orbital due to the charge transfer from O *2p* to Co *e*_*g*_ orbital. To our best knowledge, this is the first time to directly identify the ligand-to-metal charge transfer (LMCT) in cathode materials using diffraction techniques^22, 23^. And new knowledge obtained about the orbital populations and electron density redistributions of Co and O atoms enables us to explicitly depict the redox reaction process during delithiation of LiCoO_2_.

## Results

### Structure characterization

**Fig. 1:**
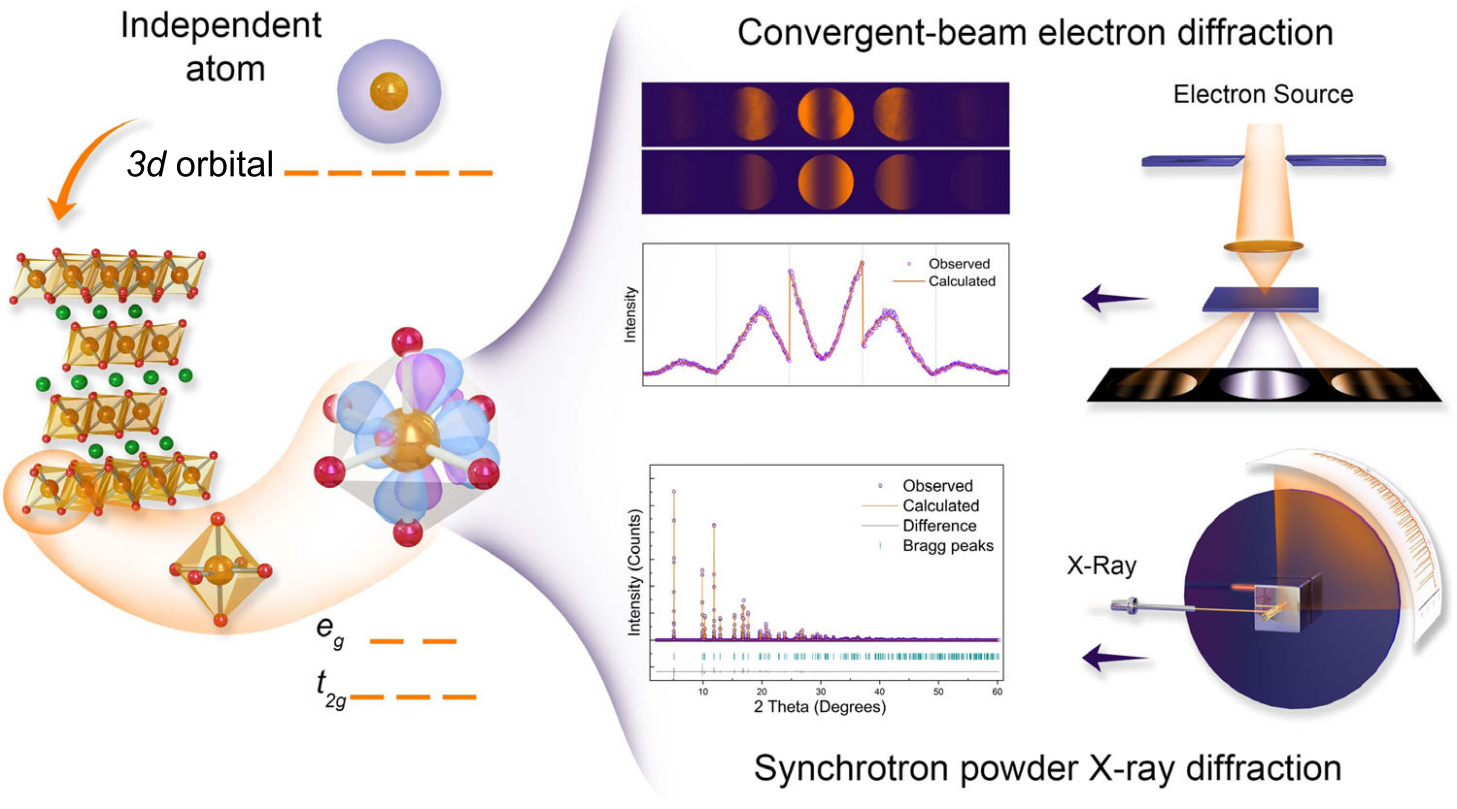
Experimental setup to obtain the three-dimensional electron density of LiCoO_2_. The valence electron distribution of a free independent transition atom (e.g., Co) is spherical, and its five *d*-orbital energy levels are degenerate. In the case of transition-metal oxides, splitting of the *d*-orbital energy levels occurs under the crystal field of polyhedra, and the redistribution of valence electrons resulting from the bonding interactions gives rise to abundant physical properties for functional materials. Using a combination of quantitative convergent-beam electron diffraction (QCBED) and synchrotron powder X-ray diffraction (SPXRD), the deformed distributions of the valence electron and orbital population can be clearly detected. In terms of SPXRD, the structure information and high-order structure factors can be extracted via Rietveld refinement. For QCBED, the low-order structure factors can be obtained using the Bloch-wave method. Through multipole refinement and quantitative topological analysis of the electron distribution, the occupancies of the *3d*-orbital can be measured.

**Fig. 2:**
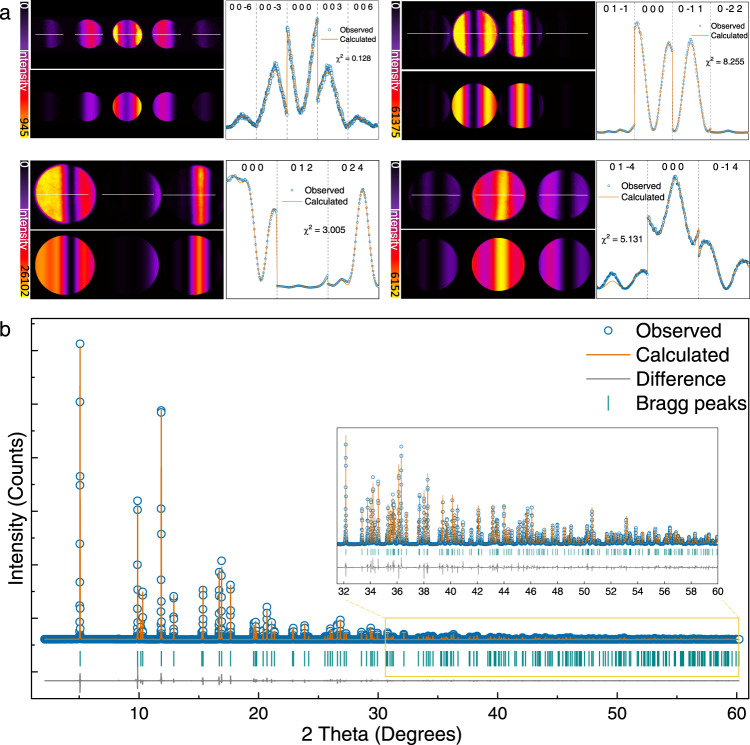
Extraction of the structure factors of pristine LiCoO_2_. **a** Measurements of the low-order structure factors from the energy-filtered CBED (003), (006), ($0\bar{1}1$), (012), and ($0\bar{1}4$) systematic rows. The first row on the left side shows the experimental CBED patterns. The second row presents the corresponding calculated patterns based on the Bloch-wave method. The intensity profiles were extracted from the experimental (dark cyan circles) and refinement results. **b** Measurements of the high-order structure factors via Rietveld refinement of the SPXRD pattern. The inset shows a magnified region ranging from 32° to 60°.

It is well known that LiCoO_2_ cathode adopts the layered α-NaFeO_2_-type structure with *R*$\bar{3}$*m* space group and undergoes successive phase transitions from H1 phase to H2, M1, H3, H1-3, O1 phase during lithium extraction^24^. In order to facilitate accurate measurement of electron density, we delicately controlled the electrochemical delithiation of single-crystalline LiCoO_2_ to get single-phase solid solutions with different SOCs at a low rate of 0.1 C (see Supplementary Fig. 1). Atomic-resolution high-angle annular dark-field scanning transmission electron microscopy (HAADF-STEM) imaging demonstrates that as-prepared Li_1-*x*_CoO_2_ displays high crystallinity and well-defined layered structure (see Supplementary Fig. 2), which is beneficial for accurate structure factor measurement. The electron density of Li_1−*x*_CoO_2_ was determined via multipole modeling of the experimental structure factors with a resolution of ${{\sin }}\theta / \lambda \approx 1.2{\AA}^{-1}$ to ensure sufficiently high resolution and precision^25^. Figure 1 presents a schematic of the entire procedure demonstrating how to map the electron-density distribution in a crystalline material and quantify the orbital population for transition-metal ions under the octahedral crystal field using complementary QCBED and high-energy SPXRD. The crystal structure parameters of Li_1−*x*_CoO_2_, including the lattice constants, atomic positions, Debye–Waller factors, and high-order structure factors, can be accurately extracted from SPXRD via Rietveld refinement (see Supplementary Figs. 3–6)^26^. To reduce the systematic errors, a low temperature (100 K) was used for all the data collection to minimize the thermal diffuse scattering and anharmonicity that contribute to the background and high-order Bragg reflections, and, finally, to enhance the signal-to-noise ratio of the X-ray diffraction data^27^. In the meantime, the low-order structure factors were accurately measured via QCBED at 100 K to ensure that the measured X-ray and electron structure factors have nearly the same temperature factors. Besides, we carefully calibrated the beam current for the CBED experiment and evaluated the beam damage, both CBED patterns and electron energy loss spectra showed that the electron dose used in our experiment would not induce the atomic and electronic structures changes at 100 K (Supplementary Figs. 9–10 and Supplementary Tables 1–2). As for the low-order structure factor measurement, the initial structure parameters obtained via Rietveld refinement from SPXRD were employed for the Bloch-wave simulation, in which the thickness, beam direction, and structure factors were treated as refinable parameters. The refinement was made by comparing the experimental intensity profile across CBED systematic rows with the calculated intensity using a goodness-of-fit criterion^28^. It should be noted that these experimental structure factors are independent of the employed simulation models^29^. Figure 2 presents the five low-order structure factor measurements for pristine LiCoO_2_, including (003), ($0\bar{1}1$), (006), (012), and ($0\bar{1}4$), which show good agreement between the experimental and calculated intensities. All of the refined low-order structure factors at different SOCs are listed in supplementary Tables 3–6.

Atom-centered multipole expansion based on the spherical harmonic functions has proven successful in describing the real-space nonspherical electron density^30^, in which the electron density of each atom is described as follows:

1 $$\rho \left({{{{{\boldsymbol{r}}}}}}\right)={\rho }_{c}+{P}_{v}{\kappa }^{3}{\rho }_{v}\left(\kappa r\right)+\mathop{\sum }\limits_{l=0}^{{l}_{{\max }}}{{\kappa }^{{\prime} }}^{3}{R}_{l}\left(\kappa^{\prime} r\right)\mathop{\sum }\limits_{m=0}^{l}{P}_{{lm}\pm }{d}_{{lm}\pm }(\theta,\phi )$$

where ${\rho }_{c}$ and ${\rho }_{v}$ denote the core and valence electron densities, respectively; ${P}_{v}$ and ${P}_{{lm}\pm }$, the population parameters of the valence electron density and the spherical harmonic density (${d}_{{lm}\pm }$), respectively; $\kappa$ and $\kappa {\prime}$, valence-shell contraction–expansion parameters; and ${R}_{l}$, the radial function. This method implicitly assigns each density fragment to the centered nucleus. Therefore, the shape of the observed electron density can be flexibly fitted by a sum of nonspherical pseudo-atomic densities. These consist of a spherical-atom (or ion) electron density obtained from multiconfiguration Dirac–Fock calculations with variable orbital occupation factors to allow for charge transfer and a small nonspherical part, in which local symmetry-adapted spherical harmonic functions were used^28^.

### Electron-density distribution and orbital populations in Li_1-*x*_CoO_2_

Bader’s quantum theory of atoms in molecules (QTAIM) and deformation densities are often used to analyze the chemical bonding interactions and charge transfer in crystals after obtaining the real-space electron-density distribution^31, 32^. QTAIM can provide a unique definition of the bonding interactions using the bond critical point (BCP), where the electron density is maximum in two directions (perpendicular to the bond) and minimum in one direction (parallel to the bond). To quantify the Co–O bonding interactions of Li_1−*x*_CoO_2_ during charging, Bader’s topological analysis was conducted based on the multipole modeling of electron density^25^. The electron-density topological analysis of the BCPs is illustrated in Table 1, which indicates that the Co–O interaction is a closed-shell interaction for the entire charging process^33^, with positive$\,{\nabla }^{2}\rho ({r}_{c})$ and $\left|{\lambda }_{1}\right|/{\lambda }_{3}\ll 1$. However, the relatively large $\rho ({r}_{c})$ suggests a covalent component in Li_1−*x*_CoO_2_, and consequently, the Co–O bond is partially covalent. This may be caused by the high electron density at the positions of the Co nuclei, which results in a sharp decrease along the Co–O bond path and a large curvature (${\lambda }_{3}$) at the BCP^34^.

**Table 1:** Topological analysis of the bond critical points along the Co–O bond in Li_1−*x*_CoO_2_

Parameters	LiCoO_2_	Li_0.6_CoO_2_	Li_0.4_CoO_2_	Li_0.3_CoO_2_
$\rho$	0.5982	0.4732	0.6817	0.6151
${\nabla }^{2}\rho$	10.49	15.79	13.43	15.43
$\nabla \rho$	$4.111 \times {10}^{-6}$	$7.982\times {10}^{-6}$	$1.250\times {10}^{-6}$	$2.896\times {10}^{-6}$
$\left|{\lambda }_{1}\right|/{\lambda }_{3}$	0.17212	0.0602	0.2868	0.0732

$\rho$, $\nabla \rho$, and$\,{\nabla }^{2}\rho$ denote the electron density, its gradient, and the Laplacian, respectively. The Hess eigenvectors are defined by the diagonalization of the symmetric matrix of the nine second derivatives of $\rho$. ${\lambda }_{1}$ and ${\lambda }_{3}$ denote the Hess eigenvalues perpendicular and parallel to the bond path at the critical point, respectively.

**Fig. 3:**
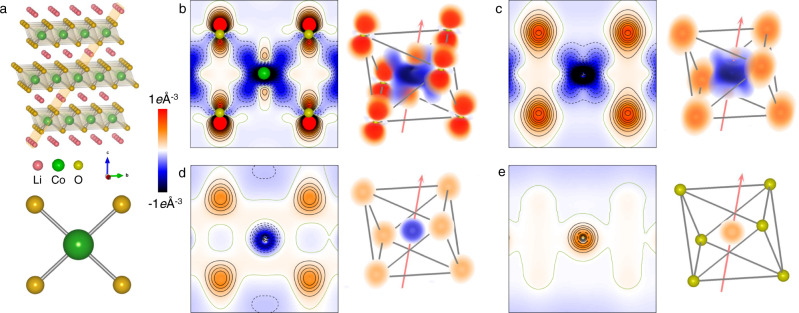
Electron density evolution of the Co–O and O–O interactions. **a** Structural model of LiCoO_2_ with the indexed ($01\bar{4}$) plane near the [100] direction (top). CoO_4_ ($01\bar{4}$) plane of the CoO_6_ octahedra (bottom). **b**–**e** Static deformation density maps of the ($01\bar{4}$) CoO_4_ plane and corresponding three-dimensional (3D) views of **b** LiCoO_2_, **c** Li_0.6_CoO_2_, **d** Li_0.4_CoO_2_, and **e** Li_0.3_CoO_2_. The electron density is present in different colors with the color scale shown on the left. The red color region means the electron density accumulation, and the blue color region represents the electron density depletion. All of the maps use a contour interval of 0.1 *e* Å^−3^, with positive, negative, and zero contours drawn as solid black, dashed black, and solid olive-green lines, respectively. The 3D renderings of the deformation density maps for the CoO_6_ octahedra are viewed in the same direction as the top model in panel **a**. The 3D version in panel **e** can be observed more clearly in Supplementary Fig. 7. The inclined arrows show the [001] direction, and all of the 3D views use the same color scheme. For clarity, the map was plotted by a homemade program, in which a translucency factor (the alpha component in the ARGB color scheme) is used to remove most of the white background.

**Fig. 4:**
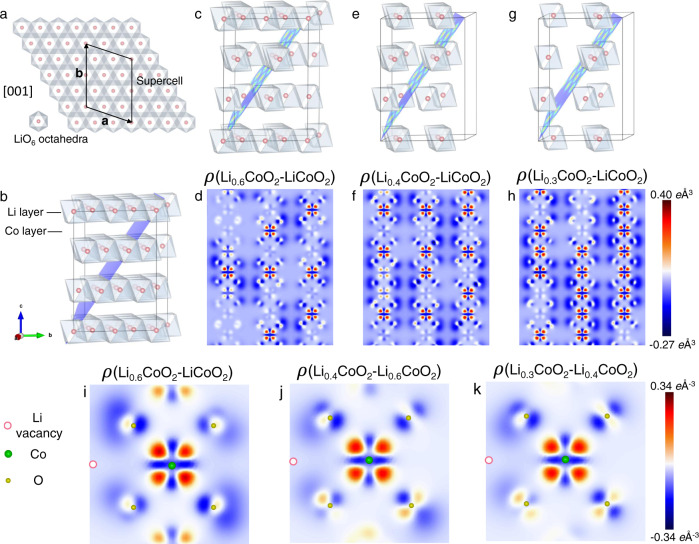
Theoretical difference electron-density maps. **a** Constructed supercell of the Li layer containing ten Li atoms along the [001] direction. **b** The *c*-axis of the supercell is the same as that of LiCoO_2_ and consists of three Li layers, three Co layers, and six O layers, with each layer containing ten atoms. Therefore, the supercell contains 30 Li atoms, 30 Co atoms, and 60 O atoms. **c**, **e**, **g** Configurations with the lowest electrostatic energy of Li_18_Co_30_O_60_, Li_12_Co_30_O_60_, and Li_9_Co_30_O_60_, respectively. To clearly illustrate the arrangement of the Li vacancies, only the Li layers are displayed in the structure models. **d**, **f**, **h** Corresponding electron-density difference maps of Li_18_Co_30_O_60_ minus Li_30_Co_30_O_60_, Li_12_Co_30_O_60_ minus Li_30_Co_30_O_60_, and Li_9_Co_30_O_60_ minus Li_30_Co_30_O_60_ in the ($1\bar{4}4$) plane. The blue planes in panels (**b**, **c**, **e**, **g**) indicate the $\left(01\bar{4}\right)$ plane of the primary lattice. **i**–**k** Electron-density difference maps of Li_18_Co_30_O_60_ minus Li_30_Co_30_O_60_, Li_12_Co_30_O_60_ minus Li_18_Co_30_O_60_, and Li_9_Co_30_O_60_ minus Li_12_Co_30_O_60_ in the ($1\bar{4}4$) plane, respectively. To better show electron density redistribution in detail, the range of color schemes used in **i**–**k** is smaller than that of **d**, **f**, **h**. The red color region and blue color region represent an increase and decrease in electron density, respectively.

Deformation density, i.e., the difference between the refined multipole electron density and the reference model constructed from the superposition of the spherical isolated atoms, shows an intuitive charge accumulation and depletion in the chemical bonding regions, as well as deformation around transition metals in line with *d*-orbital level splitting under the octahedral ligand field^32^. Figure 3 exhibits the static deformation density in the ($01\bar{4}$) plane, which contains a CoO_4_ plane of the CoO_6_ octahedra. In Fig. 3b, the shapes of the Co *e*_*g*_ and *t*_*2g*_ orbitals and the O *2p* orbital under the octahedral ligand field can be clearly observed. The electron accumulation region surrounding the Co nuclei indicated by red corresponds to the *t*_*2g*_ orbital, which has lower energy compared with the *e*_*g*_ orbital (blue region). This deformation density of the pristine LiCoO_2_ shows that *3d* electrons occupy the *t*_*2g*_ orbital rather than the *e*_*g*_ orbital of Co atom in the CoO_6_ octahedra, which agrees with the *3d*-orbital populations predicted from ligand field model. As can be seen from Fig. 3b–e, it is clear that the accumulation and depletion of the valence electron density in terms of the Co *t*_*2g*_ and *e*_*g*_ orbitals become increasingly inapparent during lithium extraction, indicating that the difference between the *t*_*2g*_ and *e*_*g*_ orbital populations is getting smaller. Meanwhile, the electron density near O atoms also decreased. To verify the experimental results, a supercell consisting of 10 unit-cells with the same *c*-axis as the primary lattice (see Fig. 4a) was constructed to calculate the total electron-density distribution of Li_1−*x*_CoO_2_ using density-functional theory (DFT+*U*). Figures 4c, e, g present the arrangements of the Li vacancies with the lowest electrostatic energy. To better present the variation of the electron density of both Co and O upon charging, the total electron densities of structures with different Li contents were subtracted from that of pristine LiCoO_2_. The electron-density difference maps of Li_1−*x*_CoO_2_ minus LiCoO_2_ in Figs. 4d, f, h unambiguously show the electron density within the *e*_*g*_ orbitals and the hole density within the *t*_*2g*_ orbitals of Co atoms surrounding the Li vacancies. In addition, a hole density of the O *2p* orbital pointing towards the Co *e*_*g*_ orbital near the Li vacancies is evident. It is apparent that with an increasing concentration of Li vacancies in the system, more and more electrons are injected into the Co *e*_*g*_ orbital, which is accompanied by an increasing hole density in the Co *t*_*2g*_ and O *2p* orbitals, indicating an LMCT process in Li_1-*x*_CoO_2_ induced by Li extraction. Moreover, the difference electron-density maps of Li_0.6_CoO_2_ minus LiCoO_2_, Li_0.4_CoO_2_ minus Li_0.6_CoO_2_, and Li_0.3_CoO_2_ minus Li_0.4_CoO_2_ are illustrated in Figs. 4i–k agrees with the results in Table 2. The agreement between the theoretical calculations and the experimental measurements suggests that the combination of QCBED and SPXRD can accurately resolve the electron-density distribution and orbital occupations in crystalline solids.

**Table 2:** Co *3d*-orbital populations and the number of valence electrons of oxygen obtained from the multipole parameters

SOC	Co ${P}_{3d}$	Co ${t}_{2g}$	Co ${e}_{g}$	O ${P}_{{valence}}$
LiCoO_2_	6.50(22)	4.83(10)	1.67(12)	6.75(11)
Li_0.6_CoO_2_	6.44(27)	4.19(14)	2.25(13)	6.58(13)
Li_0.4_CoO_2_	6.66(33)	4.26(12)	2.40(45)	6.37(16)
Li_0.3_CoO_2_	7.24(29)	2.81(12)	4.43(41)	6.03(14)

In addition to the deformation densities, the populations of Co *t*_*2g*_ and *e*_*g*_ orbitals under different SOCs were calculated from the refined multipole parameters (Supplementary Table 7). The results can be made more explicit via the orbital occupation numbers as the *3d*-orbital electrons can be described in terms of atomic orbitals (${d}_{i}$)^35^,

2 $${\rho }_{3d}=\mathop{\sum }\limits_{i=1}^{5}{P}_{i}{d}_{i}^{2}+\mathop{\sum }\limits_{i=1}^{5}\mathop{\sum }\limits_{j > i}^{5}{P}_{{ij}}{d}_{i}{d}_{j}$$

where ${d}_{i}=R(r){y}_{{lm}\pm }$, $R\left(r\right)$ is the radial function; ${y}_{{lm}\pm }$, the spherical harmonic function; and $P$, the population parameter of the atomic orbital. This expression is equal to the valence part of Eq. (1). Therefore, the relationship between the *d*-orbital occupancies and the multipole population parameters can be expressed in the form of a $15\times 15$ matrix, which can be further reduced to a smaller size under different site symmetries^35^. In LiCoO_2_, the site symmetry of Co is $\bar{3}m$ with a $4\times 4$ matrix, which converts the multipole populations to the *e*_*g*_ and *t*_*2g*_ orbital occupancies^36^, as presented in Table 2. Unlike the principle in ligand field theory that the higher energy *e*_*g*_ orbital should have a zero population for Co in a fully ionic bond, the Co *e*_*g*_ orbital has 1.67 electrons and the O valence shell is not fully filled for pristine LiCoO_2_. This can be ascribed to the hybridization of the Co *e*_*g*_ orbital and the O *2p* orbital as indicated by the covalent component of the Co–O bond^37–39^. Upon charging process of LiCoO_2_, on one hand, removing Li ions takes electrons away from Co *t*_*2g*_ orbital; on the other hand, oxygen atoms also participate the charge compensation at the high potential in the form of LMCT, leading to the dramatic increased electron populations in Co *e*_*g*_ orbital for Li_0.3_CoO_2_. This process also induces the decreasing interlayer O–O spacing compared with pristine LiCoO_2_ as shown in Supplementary Table 8. Moreover, the relationship between the rehybridization and the CoO_6_ octahedra distortion is evident. Since the bond angle of O–Co–O in the CoO_4_ plane is 85.89° in the pristine LiCoO_2_ (Supplementary Table 8), it deviates from that (90°) of regular octahedra. And the anisotropic variation of the lattice parameters caused by delithiation further aggravates the distortion of the CoO_6_ octahedra. As a result, the shorter bond length of the Co–O bond is also beneficial for the rehybridization of the O *2p* and Co *e*_*g*_ orbitals (Table 2 and Supplementary Table 8). The lattice distortion and increased oxygen hole density will impair the stability of the lattice structure during delithiation, leading to undesired capacity fading at high voltages. In order to further verify experimental measurements, we performed DFT+*U* calculations. The variation trend of the electron density distribution and the valence electron orbital populations of Co and O from experimental measurements and theoretical calculations is in accordance with each other, but there still exists deviation in the exact orbital electron populations (Table 2 and Supplementary Table 9). This probably stems from the different partitioning of electron density between multipole refinement and DFT calculations, and experimentally captured LMCT. For this reason, we performed multipole refinement of LiCoO_2_ using the calculated structure factors from WIEN2K and the results were listed in Supplementary Table 10. It can be seen that the obtained orbital populations display a good agreement with experimental results of pristine LiCoO_2_, but they are slightly different from DFT+*U* calculated ones of LiCoO_2_ in Supplementary Table 10, indicating the different space partitioning of electron density between DFT+*U* calculations and experiments. In addition, oxygen redox reaction at the high potential in the form of LMCT process led to the extra increased population in Co *e*_*g*_ orbital, which may not be adequately considered in theoretical calculations^13, 39–41^.

## Discussion

In this work, we demonstrated the effectiveness of combining CBED and SPXRD to resolve the redox chemistry of Li_1-*x*_CoO_2_ during electrochemical delithiation, by directly visualizing the redistribution of valence electron density and measuring the orbital populations of Co and O atoms. We quantified the number of electrons transfer between the redox orbitals, and found that the extraction of Li ions does not simply take electrons away from the Co *t*_*2g*_ orbital and in fact, it is accompanied by an electron gain in the Co *e*_*g*_ orbital and an increasing hole density in O *2p* orbital via rehybridization. Besides, we also successfully detected obvious LMCT at high potential, i.e., in Li_0.3_CoO_2_, which originated from the increased covalence of Co–O interactions during delithiation. Based on the above findings, it can be concluded that the TM–O interaction determines the charge compensation progress during electrochemical cycling and thus influences the properties and performance of electrode materials. By changing the local chemical environment or the symmetry of the TM–O polyhedron, including doping and elemental substitutions, the interactions between the bonded atoms can be regulated to optimize the performance of electrode materials. Thus, the ability to experimentally trace the variations of the real-space electron density distribution and the orbital occupancy of cathode materials is highly important to guiding the optimization of battery performances and offering a transformational approach for creating new electrode materials.

## Methods

### Sample preparation

Single-crystalline LiCoO_2_ was bought from Alfa with a purity of 99.5%. The delithiated samples were prepared using the electrochemical method. The high-loading electrodes (loading mass of ~100 mg) were charged to different SOCs in a Swagelok cell with Li metal as the counter electrode (1 M LiPF_6_ in ethylene, dimethyl carbonate). Subsequently, the charged Swagelok cells were disassembled, and the obtained powder samples were washed three times with dimethyl carbonate before drying. All the processes were performed in an argon-filled glove box.

The transmission electron microscopy (TEM) samples were fabricated using focus ion beam (FIB) milling with an FEI Helios 600i. To prevent surface damage from ion milling, a 40-nm-thick carbon layer was deposited via thermal evaporation. On the top of the particle, a regular Pt protection layer was deposited with standard settings using an electron beam at 5 kV and 86 pA for a 300-nm thickness followed by an ion beam at 30 kV and 80 pA for a 1-μm thickness. The particle was then directly lifted to a TEM grid using a nanomanipulator. The thinning process was first performed using the FIB cleaning cross-section method (30 kV, 0.79 nA, and tilt ±2°) until the lamella thickness reached 600 nm. Further fine milling reduced the lamella thickness to 100 nm (30 kV and 80 pA), and the final polishing process was repeated by FIB at a low voltage and current (8 kV and 23 pA) until the lamella was ~35–50-nm thick. The final polishing step was carefully conducted to avoid lamella bending. To reduce the surface damage and thickness of the amorphous layer, low-energy focused Ar ion milling was conducted using a Fischione 1040 NanoMill system.

### QCBED measurements

The CBED experiments were conducted using an aberration-corrected JEOL-ARM200CF electron microscope equipped with a Gatan imaging filter and 2048 × 2048-pixel 965 CCD camera. The accelerating voltage was calibrated to be 197.50 kV by fitting the kinetically simulated CBED pattern to the experimental one taken from a Si single crystal. An energy window of 10 eV around the zero-loss peak was selected to exclude inelastically scattered electrons, which were not included in the Bloch-wave calculations. Two convergent semi-angles were chosen to obtain the systematic row patterns due to the different *d*-spacings of the sample. A custom-developed software based on Bloch-wave theory was employed to fit experimental CBED patterns to obtain the low-order structure factors.

### SPXRD measurements

Single-crystalline samples of Li_1−*x*_CoO_2_ were sealed in a Lindeman glass capillary with an internal diameter of 0.3 mm. Synchrotron powder X-ray profiles were measured at the SPring-8 BL19B2 beamline. A large Debye–Scherrer camera with an imaging plate detector was used for the data collection. The data were collected at 100 K using an N_2_ gas flow low-temperature device. The wavelength of the incident X-rays was 0.413269 Å according to the calibration with a NIST CeO_2_ standard sample. All data were collected with a sinθ/λ_max_ value of 1.67 Å^−1^.

### Scanning transmission electron microscopy (STEM) characterization

STEM imaging was performed on an aberration-corrected JEOL-ARM200CF operated at 200 kV with a convergent semi-angle of 28 mrad and a collection angle from 90 to 370 mrad for high-angle annular dark-field image acquisition.

### Electron energy loss spectroscopy (EELS)

The EELS spectra were acquired in the TEM mode with similar experimental conditions as CBED. An energy dispersion of 0.25 eV/ch and an exposure time of 0.1 s were used to collect EELS spectra before and after electron beam radiation with a radiation time of 2 s to check the beam damage.

### Theoretical calculations

We constructed a $\sqrt{7}\times 2\sqrt{3}\times 1$ supercell containing 120 atoms to calculate the electronic structure of Li_1-*x*_CoO_2_. The Li_30_Co_30_O_60_ is for pristine LiCoO_2_; Li_18_Co_30_O_60_ is for Li_0.6_CoO_2_; Li_12_Co_30_O_60_ is for Li_0.4_CoO_2_; Li_9_Co_30_O_60_ is for Li_0.3_CoO_2_. The Vienna Ab Initio Simulation Package (VASP) based on the DFT was used to calculate the electron density and electronic structures of the above LiCoO_2_, Li_0.6_CoO_2_, Li_0.4_CoO_2_, and Li_0.3_CoO_2_^42–44^. The Perdew–Burke–Ernzerhof (PBE) functional within a generalized gradient approximation (GGA) form was adopted to treat the exchange-correlation energy^45^. DFT+*U* (*U* = 3.32 eV) was used to correct the self-interaction error of conventional DFT for correlated *d* electrons In Li_1-*x*_CoO_2_. A plane wave representation for the wave function with cut-off energy of 500 eV was applied. Geometric optimizations were performed using conjugate gradient minimization until all forces acting on the ions were less than 0.01 eV/Å per atom. The K-point mesh with a spacing of *ca*. 0.03 Å^−1^ was adopted. The magnetism moments for LiCoO_2_, Li_0.6_CoO_2_, Li_0.4_CoO_2_, and Li_0.3_CoO_2_ are optimized to 0, 0.4, 0.6, and 0.7 μ_B_/Co.

WIEN2k using a fully-potential linear augmented plane-wave method was adopted to obtain the structure factors of pristine LiCoO_2_ via Fourier transformation of the theoretical electron density^46, 47^. The structure parameters of LiCoO_2_ were adopted from experimental data. The muffin-tin radii for Li, Co, and O were chosen as 1.76, 1.93, and 1.67 Bohr. The PBE-GGA was adopted as the exchange-correlation functionals, and the selected energy and the energy convergence criterion were set to be −6.0 Ry and 10^−5^ eV, respectively. Totally 90 k-points were generated and automatically distributed in the irreducible wedge of the Brillouin zone by the program.

## Supplementary information

Supplementary Information

## Data Availability

All data supporting this study and its findings are available within the article, its Supplementary Information. All raw data generated during the current study are available from the corresponding authors upon request.
