# Author Correction: Real-space measurement of orbital electron populations for Li_1-x_CoO_2_

**DOI:** 10.1038/s41467-022-34070-6

**Published:** 2022-10-21

**Authors:** Tongtong Shang, Dongdong Xiao, Fanqi Meng, Xiaohui Rong, Ang Gao, Ting Lin, Zhexin Tang, Xiaozhi Liu, Xinyan Li, Qinghua Zhang, Yuren Wen, Ruijuan Xiao, Xuefeng Wang, Dong Su, Yong-Sheng Hu, Hong Li, Qian Yu, Ze Zhang, Vaclav Petricek, Lijun Wu, Lin Gu, Jian-Min Zuo, Yimei Zhu, Ce-Wen Nan, Jing Zhu

**Affiliations:** 1 Beijing National Laboratory for Condensed Matter Physics, Institute of Physics, Chinese Academy of Sciences, Beijing, 100190 P. R. China; 2 School of Physical Sciences, University of Chinese Academy of Sciences, Beijing, 100049 China; 3 Songshan Lake Materials Laboratory, Dongguan, 523808 P. R. China; 4 State Key Lab of New Ceramics and Fine Processing, School of Materials Science and Engineering, Tsinghua University, Beijing, 100084 P. R. China; 5 School of Materials Science and Engineering, University of Science and Technology Beijing, Beijing, 100083 P. R. China; 6 Department of Materials Science and Engineering, Center of Electron Microscopy and State Key Laboratory of Silicon Materials, Zhejiang University, Hangzhou, 310027 P. R. China; 7 Institute of Physics, Academy of Sciences of the Czech Republic, Praha, 180 40 Czech Republic; 8 Condensed Matter Physics and Materials Science Division, Brookhaven National Laboratory, Upton, New York 11973 USA; 9 Department of Materials Science and Engineering, University of Illinois at Urbana Champaign, 1304 W Green St, Urbana, 61801 USA; 10 Beijing National Center for Electron Microscopy, Laboratory of Advanced Materials, Department of Materials Science and Engineering, Tsinghua University, Beijing, 100084 P. R. China

**Keywords:** Imaging techniques, Electronic properties and materials

Correction to: *Nature Communications* 10.1038/s41467-022-33595-0, published online 03 October 2022

The original version of this Article contained an error in the Acknowledgements, in which a funding agent was omitted. The correct version contains ‘L. Wu and Y. Zhu were supported by the U.S. Department of Energy, Office of Basic Energy Science, Division of Materials Science and Engineering, under Contract No. DE-SC0012704.’ This has been corrected in both the PDF and HTML versions of the Article.

