# Supplementary material for: Real-space measurement of orbital electron populations for Li1-xCoO2
Source: Nat Commun. 2022 Oct 3;13:5810. doi: 10.1038/s41467-022-33595-0 (PMC9530229; doi:10.1038/s41467-022-33595-0)
Supplement: Supplementary file 1 — Supplementary Information [file 41467_2022_33595_MOESM1_ESM.pdf]

Supplementary Information

Real-space measurement of orbital electron populations for $Li_{1-x}CoO_2$

Tongtong Shang[1,2]†, Dongdong Xiao[1,3]†, Fanqi Meng[4]†, Xiaohui Rong[1], Ang Gao[1,2], Ting Lin[1,2], Zhexin Tang[1,2], Xiaozhi Liu[1,2], Xinyan Li[1,2], Qinghua Zhang[1], Yuren Wen[5], Ruijuan Xiao[1,2], Xuefeng Wang[1,2], Dong Su[1,2], Yong-Sheng Hu[1,2], Hong Li[1,2], Qian Yu[6], Ze Zhang[6], Vaclav Petricek[7], Lijun Wu[8]*, Lin Gu[1,2,3]*, Jian-Min Zuo[9], Yimei Zhu[8], Ce-Wen Nan[4], Jing Zhu[10]

Affiliations:

[1]Beijing National Laboratory for Condensed Matter Physics, Institute of Physics, Chinese Academy of Sciences, Beijing 100190, P. R. China

[2]School of Physical Sciences, University of Chinese Academy of Sciences, Beijing 100049, China

[3]Songshan Lake Materials Laboratory, Dongguan 523808, P. R. China

[4]State Key Lab of New Ceramics and Fine Processing, School of Materials Science and Engineering, Tsinghua University, Beijing 100084, P. R. China

[5]School of Materials Science and Engineering, University of Science and Technology Beijing, Beijing 100083, P. R. China

[6]Department of Materials Science and Engineering, Center of Electron Microscopy and State Key Laboratory of Silicon Materials, Zhejiang University, Hangzhou 310027, P. R. China

[7]Institute of Physics, Academy of Sciences of the Czech Republic, Praha 180 40, Czech Republic

[8]Condensed Matter Physics and Materials Science Division, Brookhaven National Laboratory, Upton, New York 11973, USA

[9]Department of Materials Science and Engineering, University of Illinois at Urbana Champaign, 1304 W Green St., Urbana 61801, USA

[10]Beijing National Center for Electron Microscopy, Laboratory of Advanced Materials, Department of Materials Science and Engineering, Tsinghua University, Beijing 100084, P. R. China

†Tongtong Shang, Dongdong Xiao and Fanqi Meng contributed equally to this work.

*Corresponding authors: ljwu@bnl.gov; l.gu@iphy.ac.cn

Supplementary Text

1. Mapping the electron density

The refinement and extraction of low-order structure factors were performed using a custom-developed software[1]. The refined parameters are electron-beam incident direction, sample thickness, and structure factors. To avoid very large matrices and a reasonable accuracy for the Bloch wave theory, beams were selected according to certain criteria[2]. We chose 2.6 for g_{max}, 3.0 for $2KS_{gmax}$, 0.005 for $\left|\frac{U_{max}}{2KS_g}\right|$.

Using the Mott-Bethe formula we can convert electron structure factors to X-ray structure factors, and replace the corresponding structure factors to perform multipole refinement. The refined low-order electron and X-ray structure factors of $Li_{1-x}CoO_2$ are listed in Supplementary Tables 3–6. The high-order X-ray structure factors are mainly determined by the atomic position, atomic displacement parameters, and core electrons. It can be refined and extracted using Rietveld refinement from SPXRD.

All Rietveld refinement and multipole refinement were performed in JANA2006 software with scattering factors derived from Slater-type orbitals (STO) atomic relativistic wavefunctions[3]. The Rietveld refinements are shown in Supplementary Figures 3–6. To accurately extract the high-order structure factors, the resolution of the data was cut off from 1.67 $Å^{-1}$ to 1.2 $Å^{-1}$, beyond which the overlap between diffraction peaks was severe. Since the multipole model is based on spherical harmonic density functions centered around each atomic nucleus[4], the limit l_{max} = 4 was used for truncation of the multipole expansion at the hexadecapole level. The functions allowed for a particular site are symmetrically restricted. For both Co ($\bar{3}m$) and O ($3m$), the *z*-axis is parallel to the *3*-fold axis [001] direction, and the *y* axis is perpendicular to the mirror plane, i.e., alone the [010] direction. Several constraints were imposed on the multipole refinement: the valence population of Li ion was fixed at $P_v = 0$, $\kappa_{[Li]} = 1$ and the electron deformation density of Li was not refined. For Co, only the seven *3d* electrons were considered as valence electrons. The two *4s* electrons were treated as core electrons, since the *4s* electrons contribute very little to the scattering factors[5]. The topological electron density analysis of the obtained total electron densities based on Bader's quantum theory of atoms in molecules was also calculated using JANA2006[3]. The results for the bond critical points are shown in **Table 1**.

2. Co *3d*-orbital populations

In $Li_{1-x}CoO_2$, the CoO_6 octahedra is trigonally distorted. The *3d* electron density can be expressed by[5]

$$\rho_{3d} = [R(r)]^2 \left\{P_1(a_1)^2 + (1/2)P_2[(e_{g-})^2 + (e_{g+})^2] + (1/2)P_3[(e'_{g-})^2 + (e'_{g+})^2]\right\} \qquad \text{(S1)}$$

where $R(r)$ is the radial function, (a_1), (e_{g-}) and (e_{g+}) correspond to the three t_{2g} orbitals, $(e_{g-})'$ and $(e_{g+})'$ correspond to the two e_g orbitals. P_1, P_2 and P_3 are the occupancies of the (a_1), (e_g) and $(e_g)'$, respectively.

The relationship between the occupancies of *3d* orbital and the populations of spherical density functions, which are refined from multipole modelling, is given by

$$P_1 = (1/3)P_{00} + (1/7)[(196\pi/5)^{1/2}/M_{20}]P_{20} + (3/35)[(196\pi)^{1/2}/M_{40}]P_{40}$$

$$P_2 = (2/3)P_{00} - (5/7)[(196\pi/5)^{1/2}/M_{20}]P_{20} + (6/35)[(196\pi)^{1/2}/M_{40}]P_{40} - \sqrt{2}P_4$$

$$P_3 = (1/3)P_{00} + (4/7)[(196\pi/5)^{1/2}/M_{20}]P_{20} - (9/35)[(196\pi)^{1/2}/M_{40}]P_{40} + \sqrt{2}P_4$$

(Supplementary 2)

$$P_4 = 3/(6\sqrt{2} + 1)\Big\{-(9/7)[(196\pi/5)^{1/2}/M_{20}]P_{20} + (3/7)[(196\pi)^{\frac{1}{2}}/M_{40}]P_{40} + [(63\pi/5)^{1/2}/M_{43}]P_{43+}\Big\}$$

where the P_{00}, P_{20}, P_{40}, P_{43+} are the refined populations of spherical harmonic density functions, respectively. M_{lm} denotes the ratio of normalization factors for wave functions and density functions. The Co *3d*-orbital occupancies can be calculated according to Supplementary Eq. 2.

3. Electron dose and beam damage

Cathode materials, especially in the charged states, are highly sensitive to the electron beam damage. The common methods to reduce the damage effect in TEM are minimizing the dose and cooling the specimen. In addition to cooling the specimen, we focused and tilted the specimen when irradiating one area, but recorded the data at another adjacent area to reduce the beam damage before data collection. Meanwhile, the diffraction patterns were captured as fast as possible to reduce the recording time. Due to the different d-spacings, we collected the CBED patterns with two different condenser lens (CL) apertures while keeping other conditions unchanged. For (003) and (006), a

20-um CL aperture was adapted. For other three structure factors, a 50-um CL aperture was adapted. Supplementary Table 1 show the *d*-spacing, the corresponding aperture and convergent semi-angle used to collect CBED patterns of $LiCoO_2$. All the experimental conditions for $Li_{1-x}CoO_2$ at different SOC were the same, including the convergent semi-angle. Supplementary Table 2 presents the beam currents and dose rates of the two experimental conditions.

The beam damage could induce the change of atomic and electronic structures. Accordingly, electron diffraction and electron energy loss spectroscopy (EELS) were employed to clarify whether the changes of crystal structure and electronic structure occurred during the experiments. In general, the diffraction disc of CBED contains abundant structural information of the specimen compared to the diffraction spots of parallel-beam electron diffraction (PBED), and hence it is more sensitive to the structure change[6]. Supplementary Figure 9 illustrates the $(0\bar{1}1)$ systematic row of $Li_{1-x}CoO_2$, which clearly show the changes of diffraction patterns before and after electron beam damage. If the rocking curves become crooked and blurry in the diffraction disc, it can be deduced that the structure of specimen changes. And defects in the specimens can also lead to the appearance of fringes and/or lines splitting in the diffraction disc. To obtain the accurate refined results, we selected the CBED patterns without any fringes and HOLZ line splitting. Besides, beam damage will induce large deviations of the refinement results from the experimental intensity if there exist defects in the observed sample region due to the high sensitivity of CBED pattern to structural changes.

Besides, EELS experiments of the specimen at different SOC before and after electron beam radiation were performed to detect the electronic structure changes. To make sure that the electron dose is the same for both EELS and CBED experiments, the electron beam radiation conditions for the EELS is the same as that of the CBED. As the beam current is larger for the large CL aperture, we acquired the EELS using the 50-μm aperture. If there are no changes of the electronic structures under this condition, it would be the same with the smaller CL aperture. As seen in Supplementary Figure 10, there are no obvious changes of the electronic structure after electron beam radiation on the specimen, hence we think that the electron dose used in CBED will not lead to artifacts to our experimental results.

4. DFT calculations

For pristine $LiCoO_2$, there is no need to build supercell for simulation to verify the experimental density map. In order to ensure the enough accuracy of the DFT calculations, WIEN2k with a full-potential linear augmented plane-wave method was used to calculate the electron density of $LiCoO_2$[7, 8]. The experimental lattice parameters and atomic positions were used for the calculations, and structure factors were obtained by Fourier transformation of the theoretical electron density. Multipole refinement using the theoretical structure factors gives rise to theoretical deformation electron density distributions in $(01\bar{4})$ plane, which is compared to experimental results as shown in Supplementary Figure 11. The difference between the experimental and theoretical deformation density is mainly located at the core region of the atoms, which mainly depends on high-order reflections. While the obtained high-order reflections from our experimental XRD data are still limited (g_{max} ~ 1.2 $Å^{-1}$) to refine core electron density. However, we noted that it is the valence electron parts of the Co and O atoms that significantly affect the electrochemical properties of materials. The valence electron distribution from our experimental map agrees with the theoretical calculation very well. The corresponding *3d*-orbital electron population of Co calculated from refined multipole parameters are also listed in Supplementary Table 10. The orbital electron populations from experimental measurements are close to the calculated one, demonstrating the reliability of the experimental results.

For delithiated $Li_{1-x}CoO_2$, in order to make the arrangement of Li vacancies more uniform, a supercell with 10 unit-cells and 120 atoms was constructed to simulate the configurations of $Li_{1-x}CoO_2$ with different Li contents (Supplementary Figure 12). The supercell maintains the closed-packed oxygen ABCABC framework, and enlarges the primary lattice perpendicular to the *c* axis by 10 unit-cells. (**Figs. 4a–b**) The relationship between the new basis vectors $[\boldsymbol{a}'\ \boldsymbol{b}'\ \boldsymbol{c}']$ and the primary basis vectors $[\boldsymbol{a}\ \boldsymbol{b}\ \boldsymbol{c}]$ can be obtained by:

$$[\boldsymbol{a}'\ \boldsymbol{b}'\ \boldsymbol{c}'] = [\boldsymbol{a}\ \boldsymbol{b}\ \boldsymbol{c}] \cdot R$$

$$R = \begin{bmatrix} 4 & 2 & 0 \\ -1 & 2 & 0 \\ 0 & 0 & 1 \end{bmatrix}$$

where *R* is the transformation matrix.

After constructing the superlattice, the electrostatic energy for every possible configuration were calculated and the configurations with the lowest electrostatic energy for $Li_{0.6}CoO_2$, $Li_{0.4}CoO_2$ and $Li_{0.3}CoO_2$ as the form of $Li_{18}Co_{30}O_{60}$, $Li_{12}Co_{30}O_{60}$ and $Li_9Co_{30}O_{60}$ were selected to simulate the

electron density distributions, respectively (see **Figs. 4c, e, g**). However, it is extremely time-consuming to calculate the electron density of charged states with so many atoms by WIEN2k. Instead, a projector augmented wave pseudopotential method in the VASP was used to calculate the electron density of the above configuration[9, 10, 11]. The total electron density of each configurations (**Figs. 4a, c, e, g**) was calculated. Supplementary Figures 13–18 present the density of states (DOS) of Co and O, the deformation electron density of $Li_{1-x}CoO_2$ in $(1\bar{4}4)$ plane, showing the differences between the theoretical electron density and that of the superimposed neutral atoms, as well as the band structure of constructed $Li_{1-x}CoO_2$. Here, the $(1\bar{4}4)$ plane of constructed supercell $LiCoO_2$ is the $(01\bar{4})$ plane of the actual $LiCoO_2$. It can be clearly seen that the theoretical electron density of pristine $LiCoO_2$ can well match with the experimental result. For charged $LiCoO_2$, there exists deviation between the theoretical and experimental electron density. This could be due to the fact that the DFT calculations give the local configurations with different contents of Li vacancies, while the experiments provide the averaged results of the systems. To explicitly characterize the variation of electron density and Co *3d*-orbital electron populations upon charging, we plotted the difference of electron density between the charged states and pristine $LiCoO_2$, while holding all structural parameters unchanged. The total electron density difference maps clearly demonstrated that the electrons decrease within the t_{2g} orbital and O states, and increase within the e_g orbital of Co atoms during charging, as shown in **Figs. 4d**, **4f** and **4h**. To clearly show the variation of the electron density during charging, the electron density difference maps of $Li_{18}Co_{30}O_{60}$ minus $Li_{30}Co_{30}O_{60}$, $Li_{12}Co_{30}O_{60}$ minus $Li_{18}Co_{30}O_{60}$, and $Li_9Co_{30}O_{60}$ minus $Li_{12}Co_{30}O_{60}$ are exhibited in **Figs. 4i**–**k**. It demonstrates that with the continuous extraction of Li ions from $LiCoO_2$, the electrons in t_{2g} orbital of Co and O states decrease, while the electrons in e_g orbital of Co increase. This is consistent with the results obtained from the experimental results.

In addition to the electron density maps, we also obtained the valence electron orbital populations of Co and O from the calculated results. Because the orbital electron partition depends on the coordinate system of the lattice, the coordinate system of the CoO_6 octahedron in $LiCoO_2$ is not parallel to the *XYZ* axis of the lattice. And thus, the integrated *d*-orbital electron population from PDOS is not in accordance with the experiment results. To deal with it, we constructed a cubic supercell of $Li_{16}Co_{16}O_{32}$, in which the coordinate system of the CoO_6 octahedron is in line with that of the supercell while not changing the structure of $LiCoO_2$. We constructed $Li_{16}Co_{16}O_{32}$, $Li_{10}Co_{16}O_{32}$, $Li_7Co_{16}O_{32}$ and $Li_5Co_{16}O_{32}$ for $LiCoO_2$,

$Li_{0.6}CoO_2$, $Li_{0.4}CoO_2$ and $Li_{0.3}CoO_2$, respectively, and integrated the PDOS from around -8 eV to 0 eV of Co atoms to get orbital populations, as illustrated in Supplementary Table 9.

Supplementary Figures

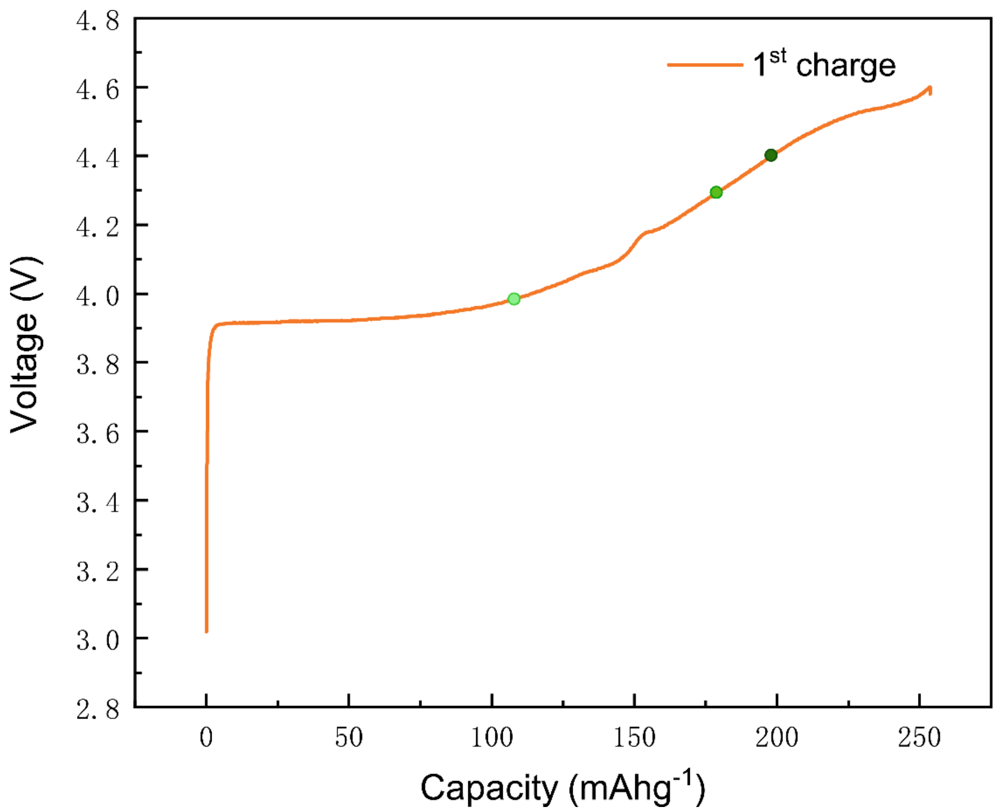

Supplementary Figure 1. Initial charging curve of $LiCoO_2$ in the voltage range of 3.0 V–4.6 V (versus Li/Li^+). The green circles from the left to the right correspond to x=0.4, 0.6, 0.7 for $Li_{1-x}CoO_2$, respectively.

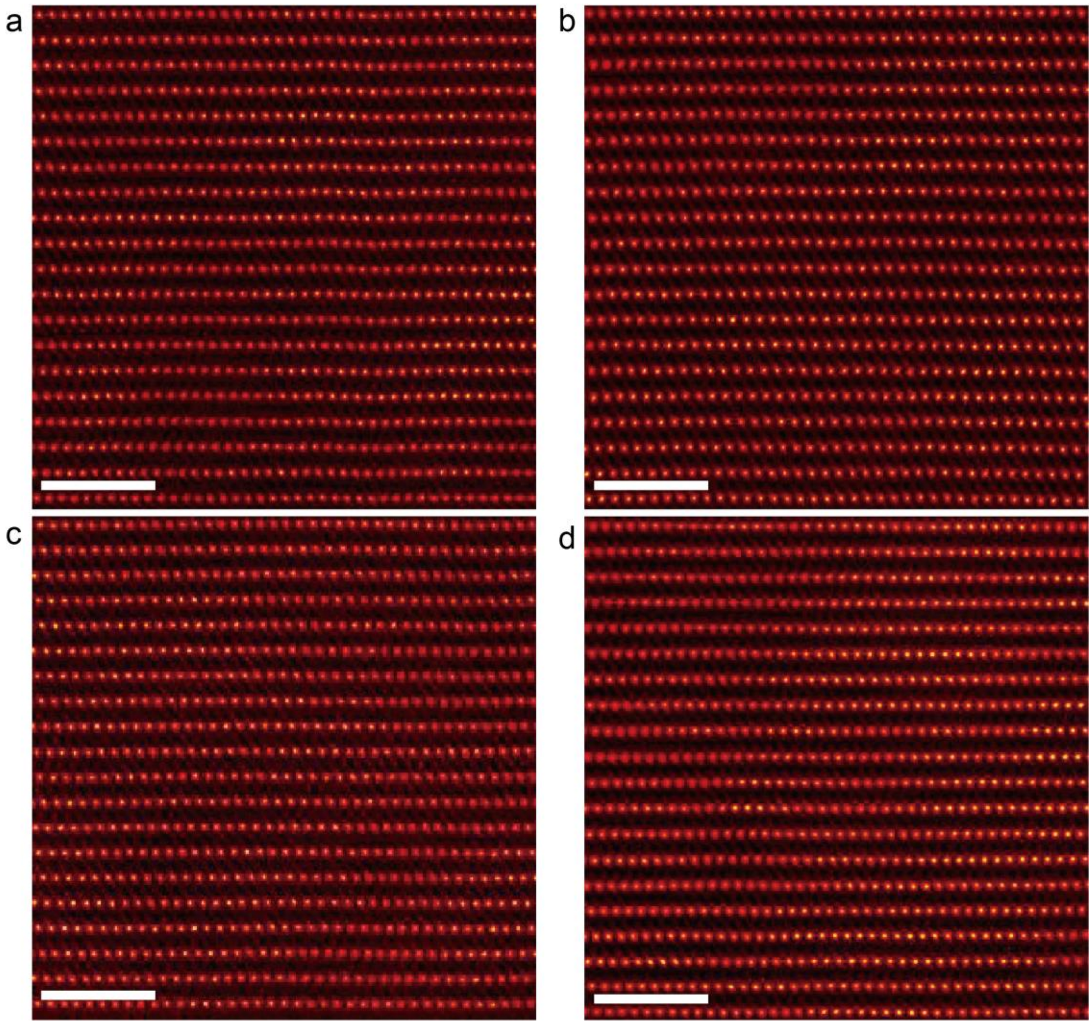

Supplementary Figure 2. Atomic-resolution HAADF-STEM images of $Li_{1-x}CoO_2$ along the [100] zone axis. **a** pristine $LiCoO_2$, **b** $Li_{0.6}CoO_2$, **c** $Li_{0.4}CoO_2$, **d** $Li_{0.3}CoO_2$. Scale bar: 2 nm

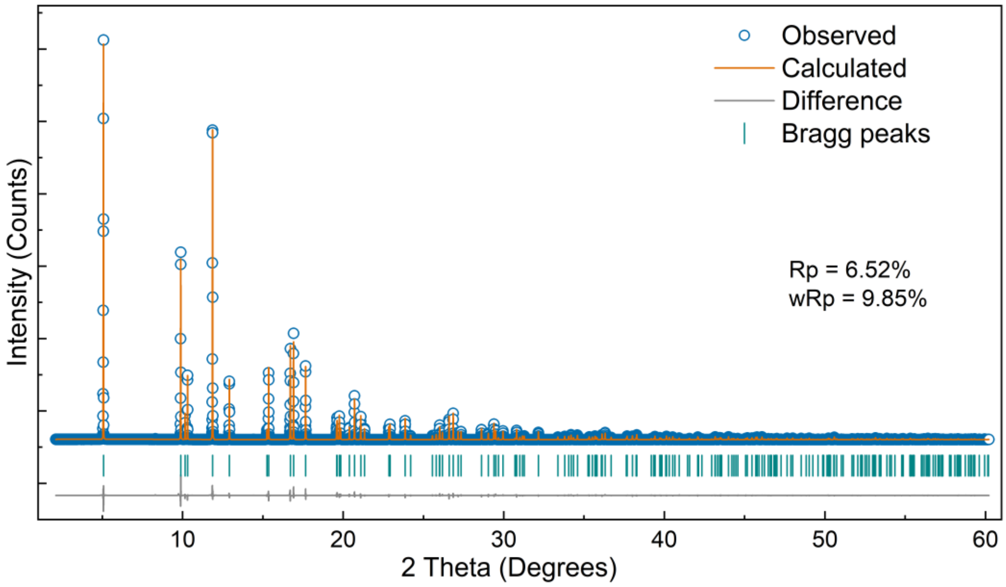

Supplementary Figure 3. Rietveld refinement for the SPXRD pattern of pristine $LiCoO_2$. There are no extra peaks, indicating the pristine $LiCoO_2$ is single phase.

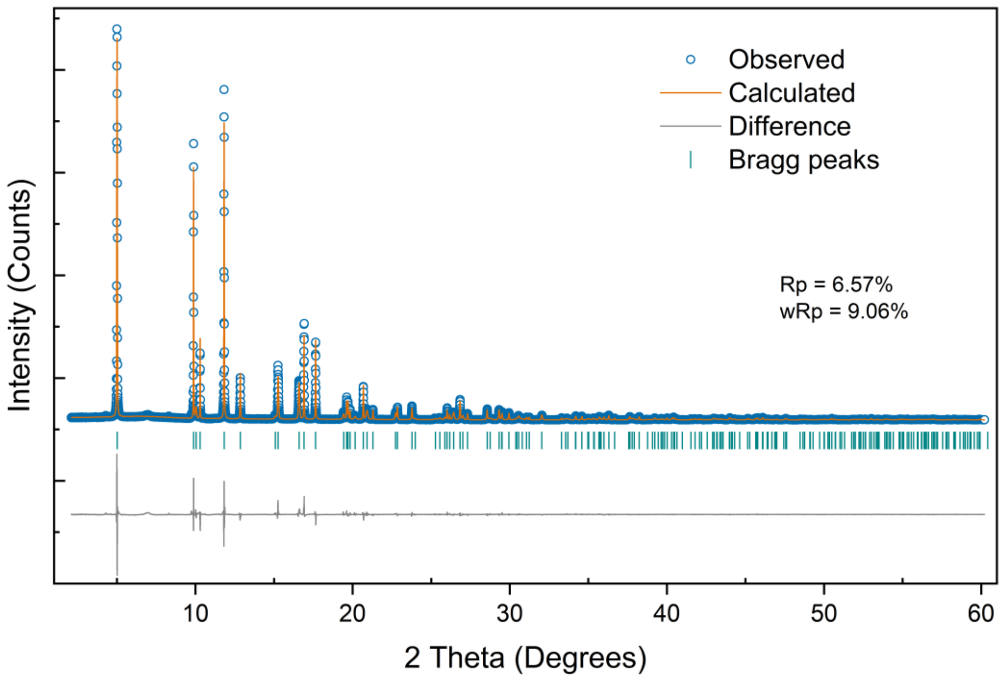

Supplementary Figure 4. Rietveld refinement for the SPXRD pattern of $Li_{0.6}CoO_2$. There are no extra peaks, indicating the delithiated $Li_{0.6}CoO_2$ is single phase.

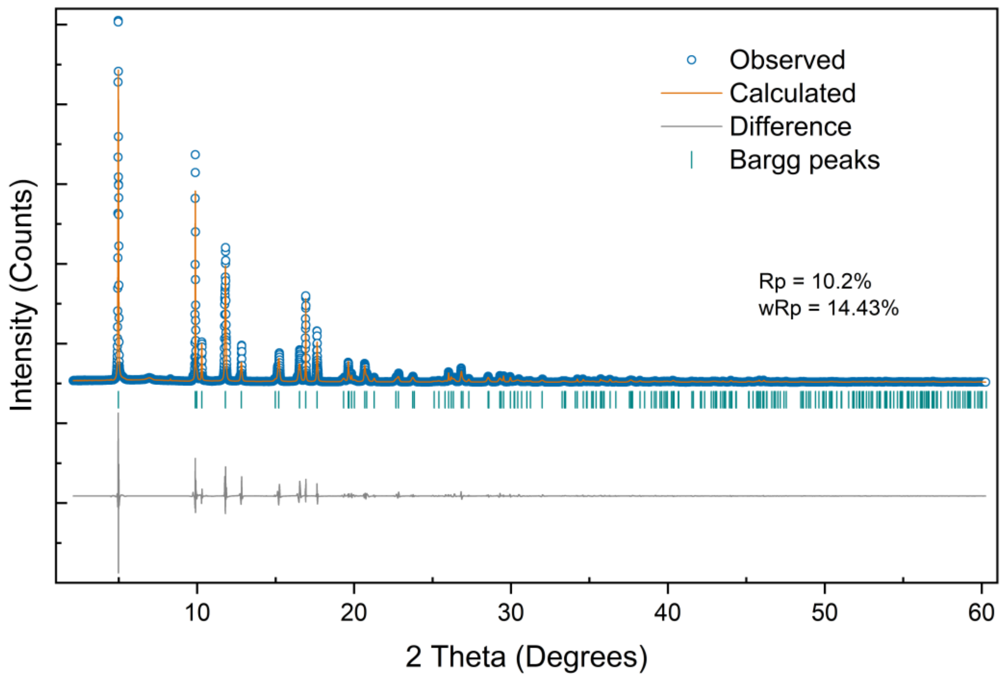

Supplementary Figure 5. Rietveld refinement for the SPXRD pattern of $Li_{0.4}CoO_2$. There are no extra peaks, indicating the delithiated $Li_{0.4}CoO_2$ is single phase.

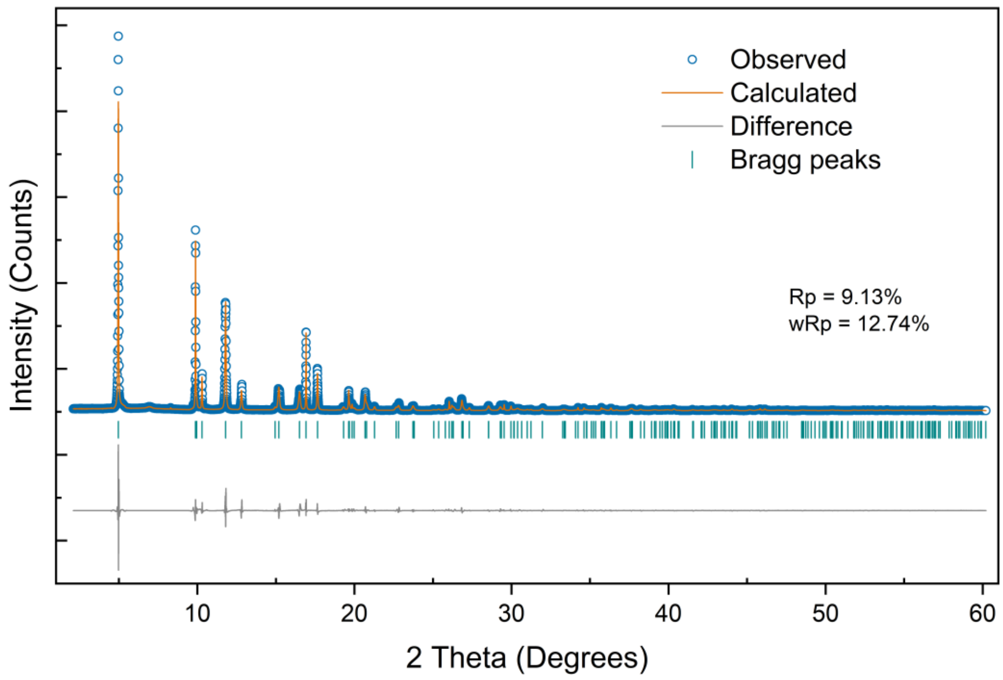

Supplementary Figure 6. Rietveld refinement for the SPXRD pattern of $Li_{0.3}CoO_2$. There are no extra peaks, indicating the delithiated $Li_{0.3}CoO_2$ is single phase.

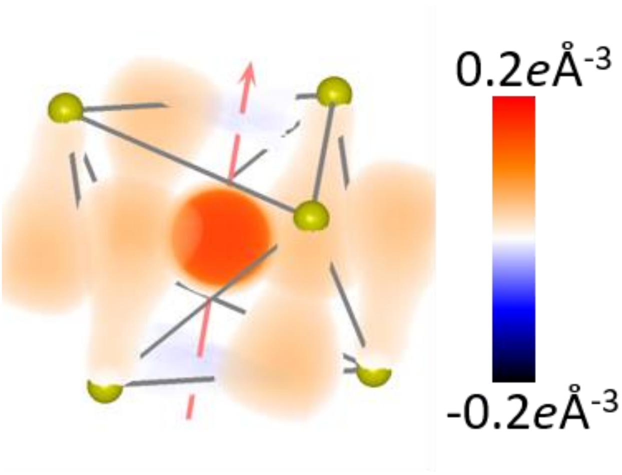

Supplementary Figure 7. 3D rendering of the static deformation density map of the CoO_6 octahedra for $Li_{0.3}CoO_2$. In this map, the color scheme ranges from -0.2 to 0.2 $\boldsymbol{e}\,Å^{-3}$ and can offer more information compared to the right panel of **Fig. 3e.**

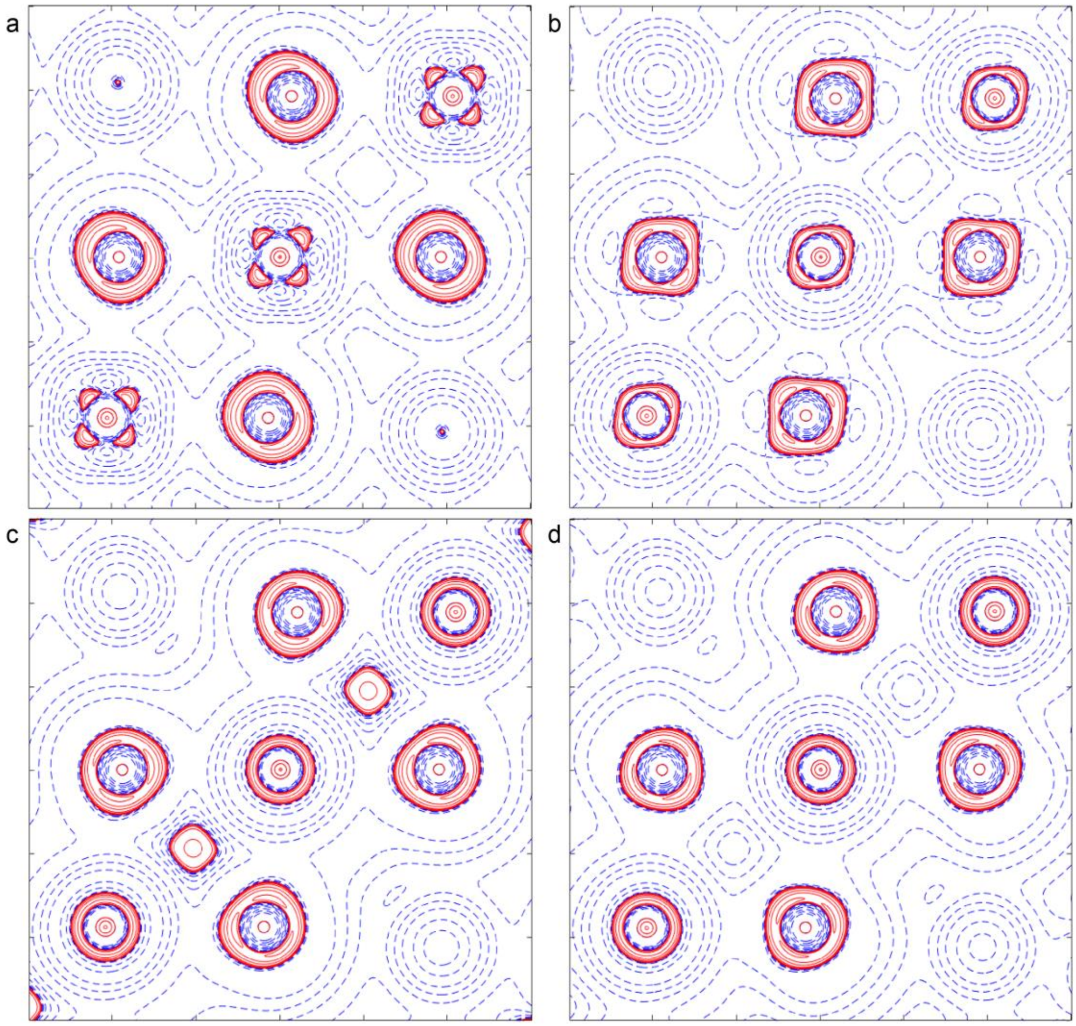

Supplementary Figure 8. Laplacian maps in the $(01\bar{4})$ plane. a $LiCoO_2$, **b** $Li_{0.6}CoO_2$, **c** $Li_{0.4}CoO_2$, **d** $Li_{0.3}CoO_2$. Contours are drawn at $\pm 2\times10n$, $\pm 4\times10n$ and $\pm 8\times10n$ e Å^{-5} (n = ± 3, ± 2, ± 1, 0), with solid red and dotted blue lines for positive and negative values, respectively.

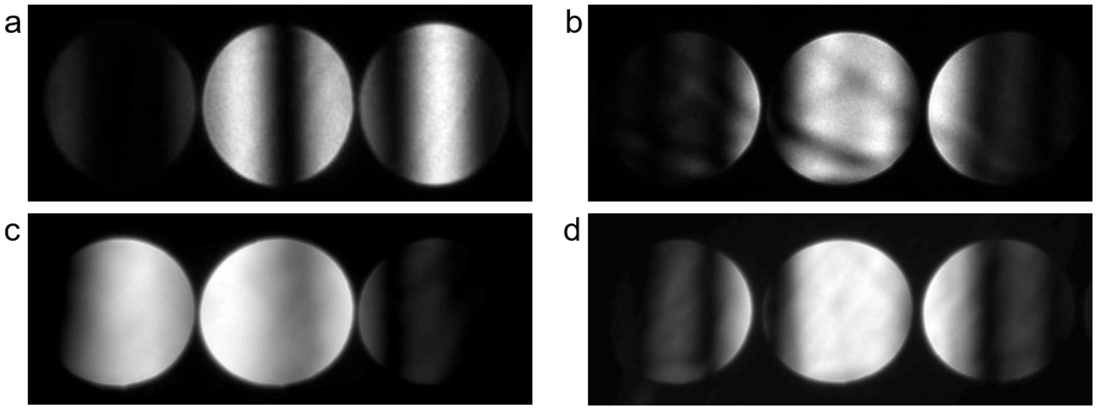

Supplementary Figure 9. Comparison of $(0\bar{1}1)$ systematic row CBED patterns of $Li_{1-x}CoO_2$ with and without structural changes. **a** is the diffraction pattern without structure change. The messy contrast and crooked rocking curves in **b-c**, and the sideling fringes in **d** indicate the structure changes during beam radiation.

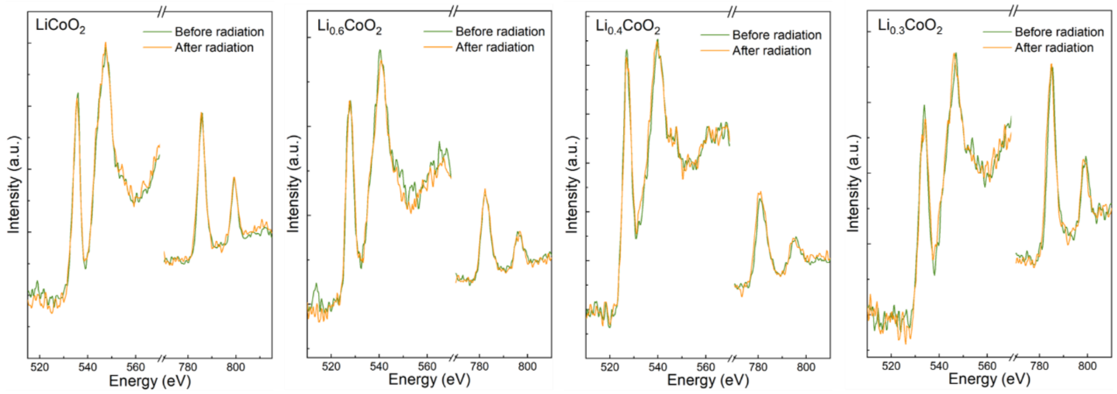

Supplementary Figure 10. EELS spectra of $Li_{1-x}CoO_2$ before and after radiation. The experimental detail is in the Method.

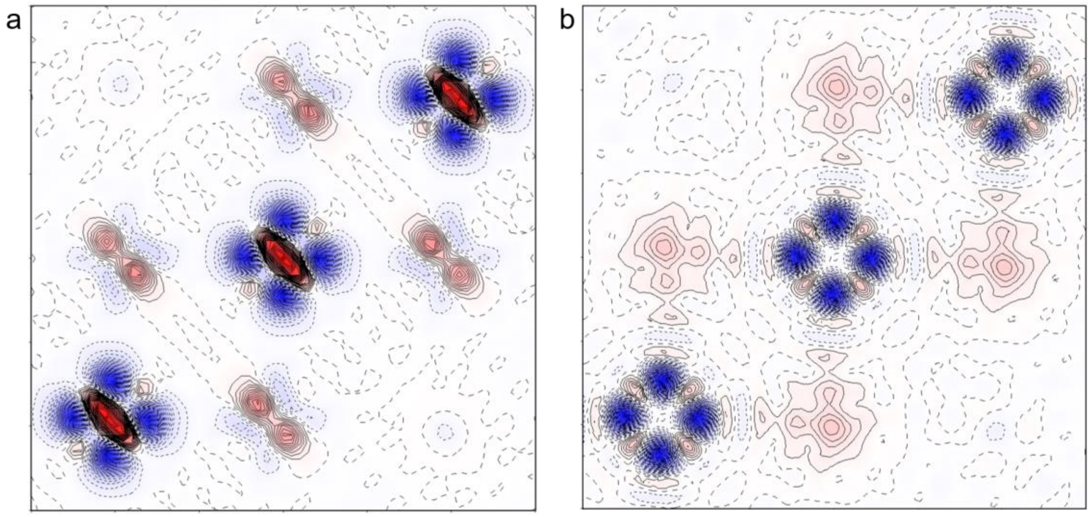

Supplementary Figure 11. Comparison between (a) experimental and (b) theoretical deformation densities in $(01\bar{4})$ plane of pristine $LiCoO_2$. The map was drew using JANA2006[3]. The contour interval is $0.1\ e\ Å^{-3}$, with positive and negative contours drawn as solid red and dotted blue lines, respectively.

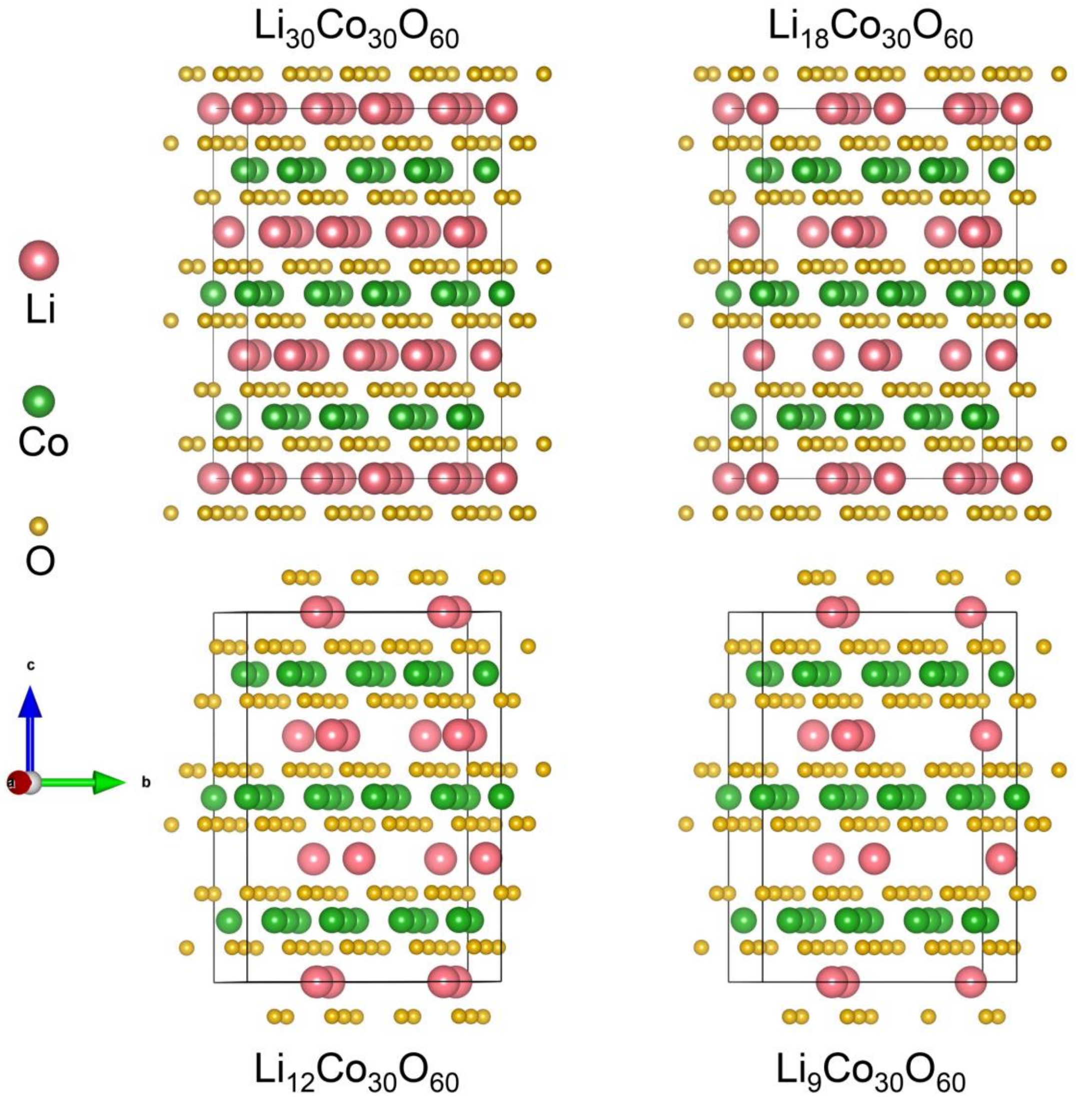

Supplementary Figure 12. Crystal structures of the constructed $Li_{1-x}CoO_2$. Each supercell contains 10 unit cells.

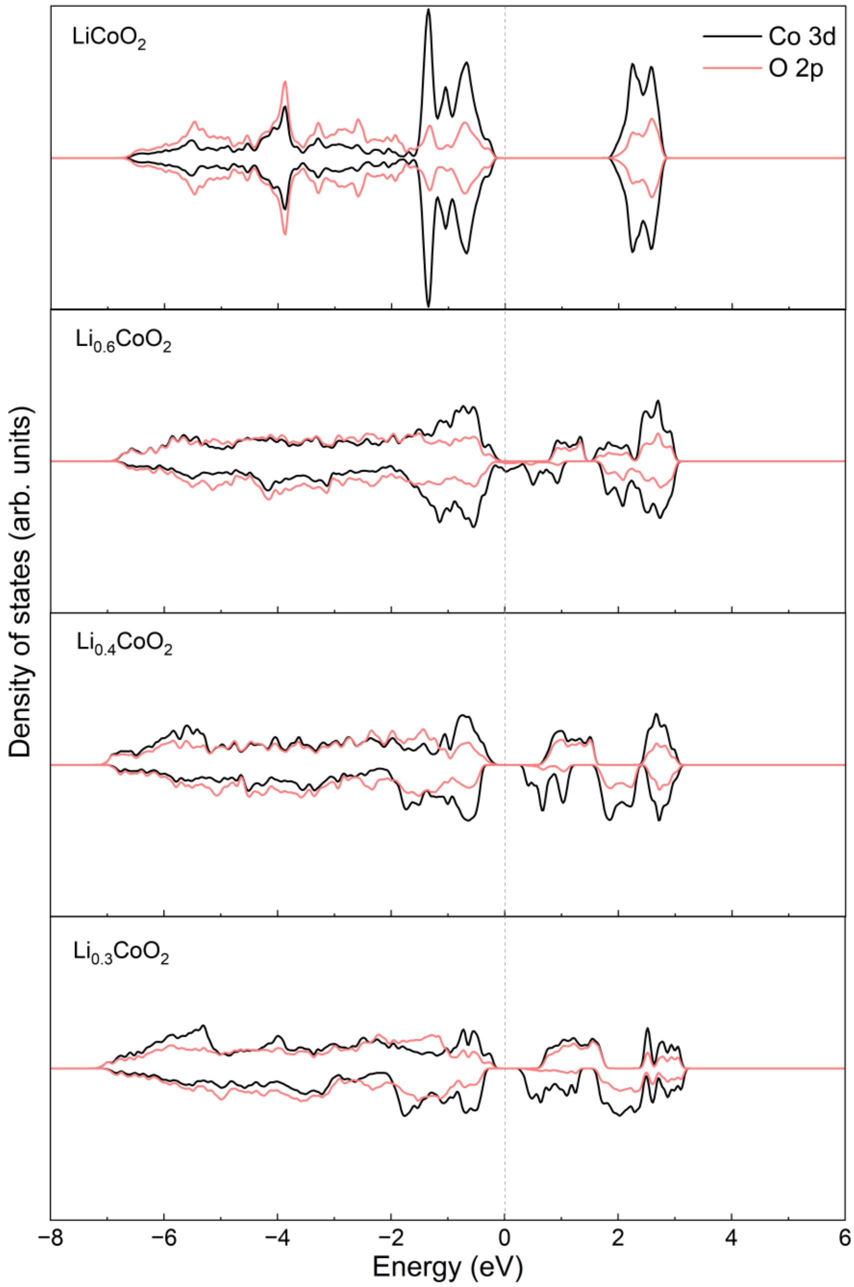

Supplementary Figure 13. Calculated DOS of $Li_{1-x}CoO_2$. These results are from the constructed supercells in Supplementary Figure 12.

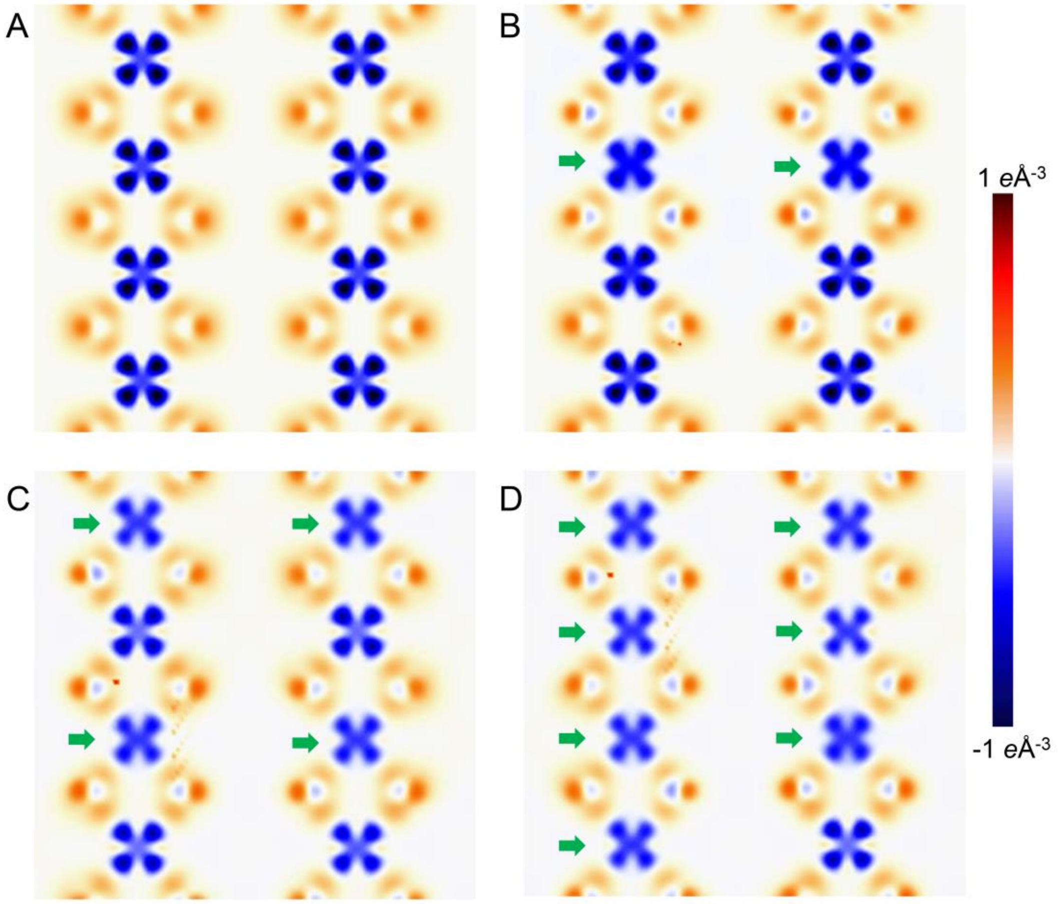

Supplementary Figure 14. Theoretical deformation density of $Li_{1-x}CoO_2$. The deformation electron density is difference between the theoretical electron density and independent atom model (IAM) for a LiCoO2, b Li0.6CoO2, c Li0.4CoO2 and d Li0.3CoO2 in ($\mathbf{1\bar{4}4}$) plane. The changed electron densities are pointed by green arrows.

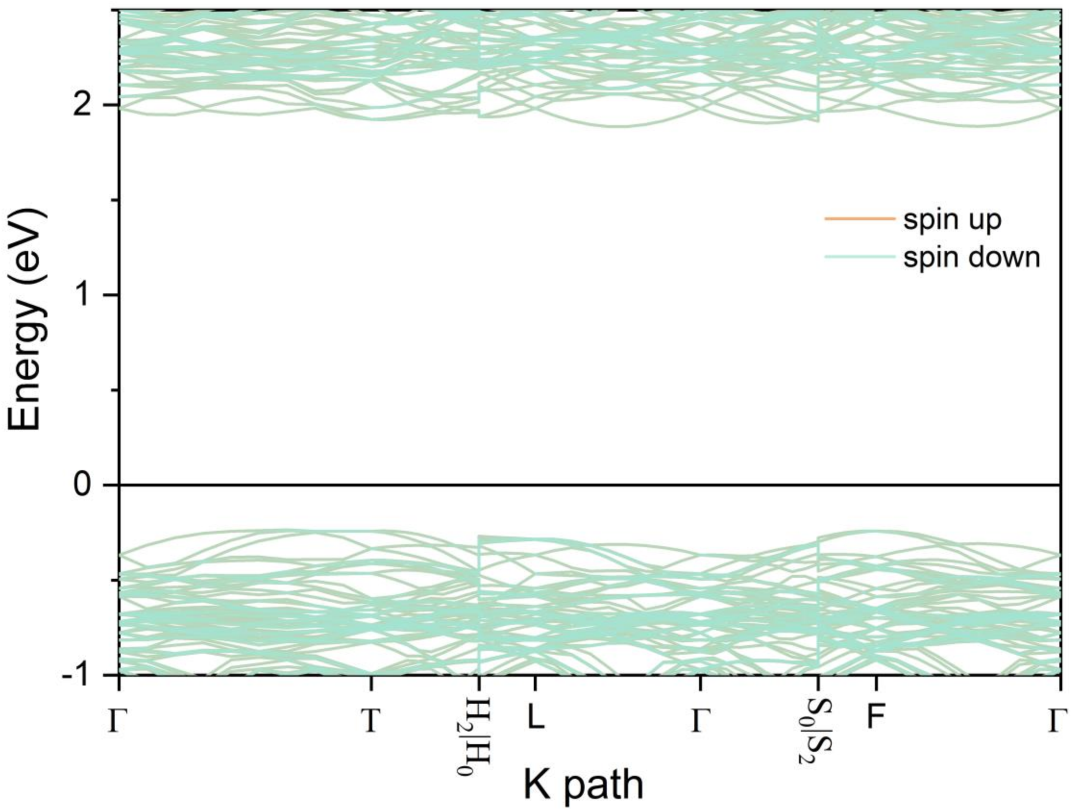

Supplementary Figure 15. Calculated band structure of $LiCoO_2$. This band structure is the calculated result from constructed supercell $Li_{30}Co_{30}O_{60}$ in Supplementary Figure 12.

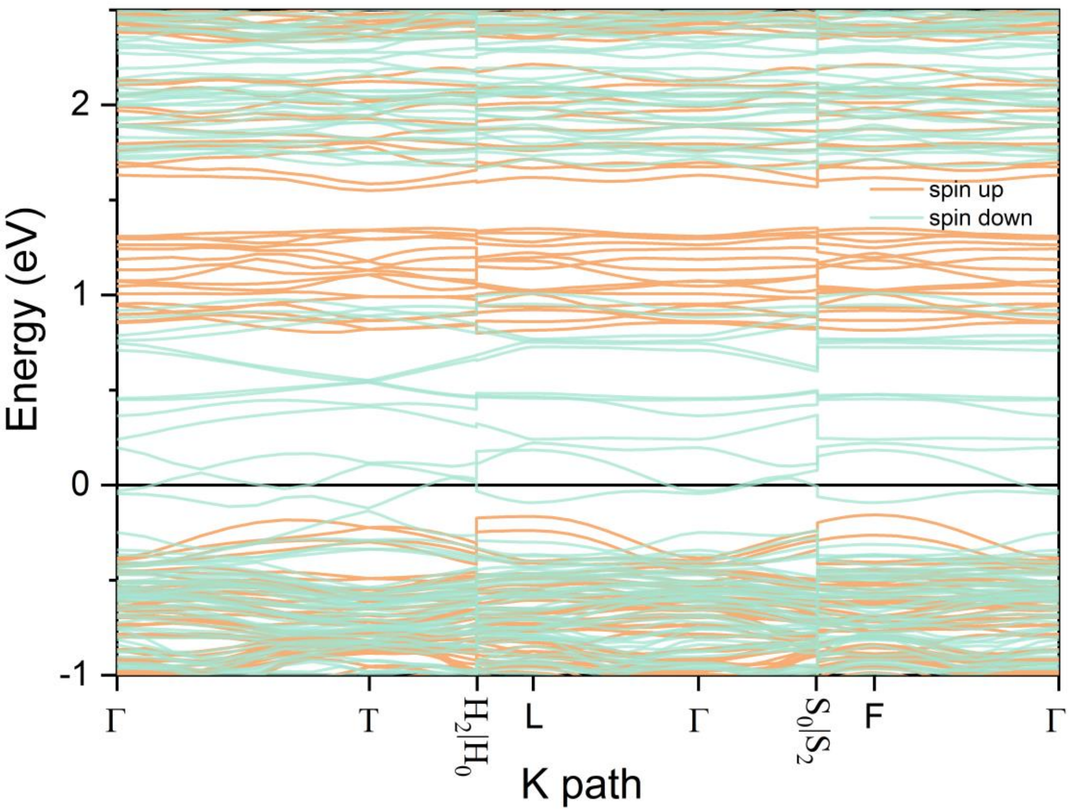

Supplementary Figure 16. Calculated band structure of $Li_{0.6}CoO_2$. This band structure is the calculated result from constructed supercell $Li_{18}Co_{30}O_{60}$ in Supplementary Figure 12.

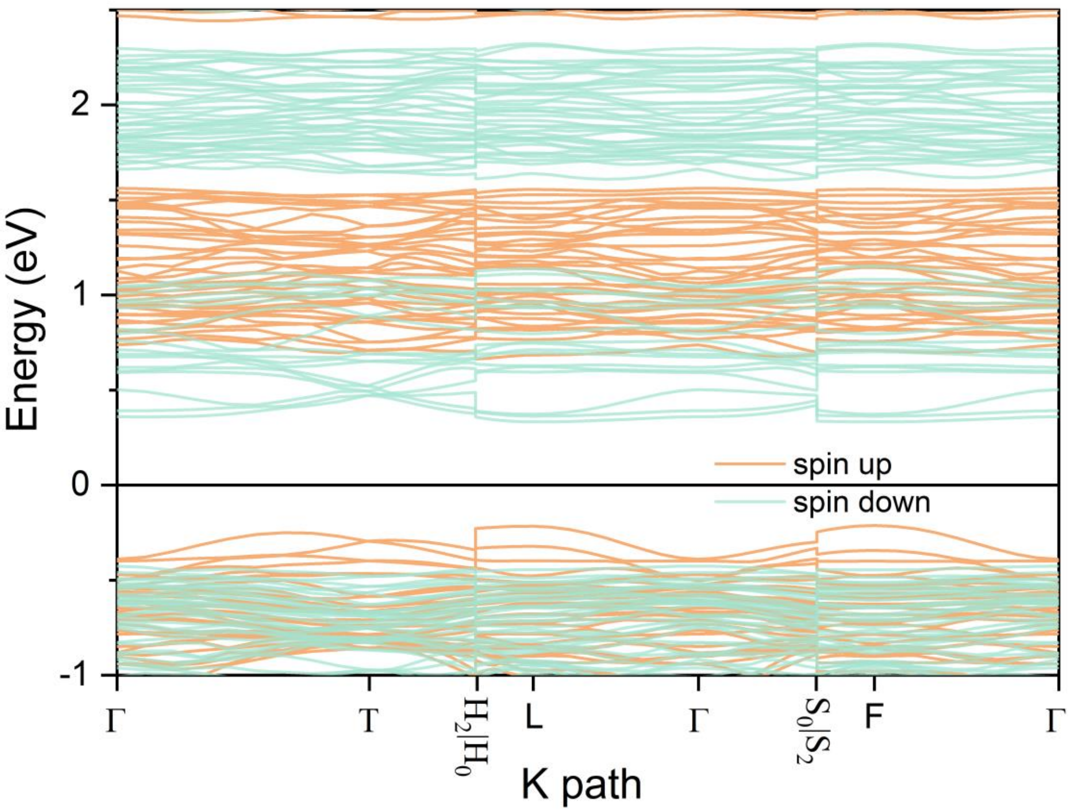

Supplementary Figure 17. Calculated band structure of $Li_{0.4}CoO_2$. This band structure is the calculated result from constructed supercell $Li_{12}Co_{30}O_{60}$ in Supplementary Figure 12.

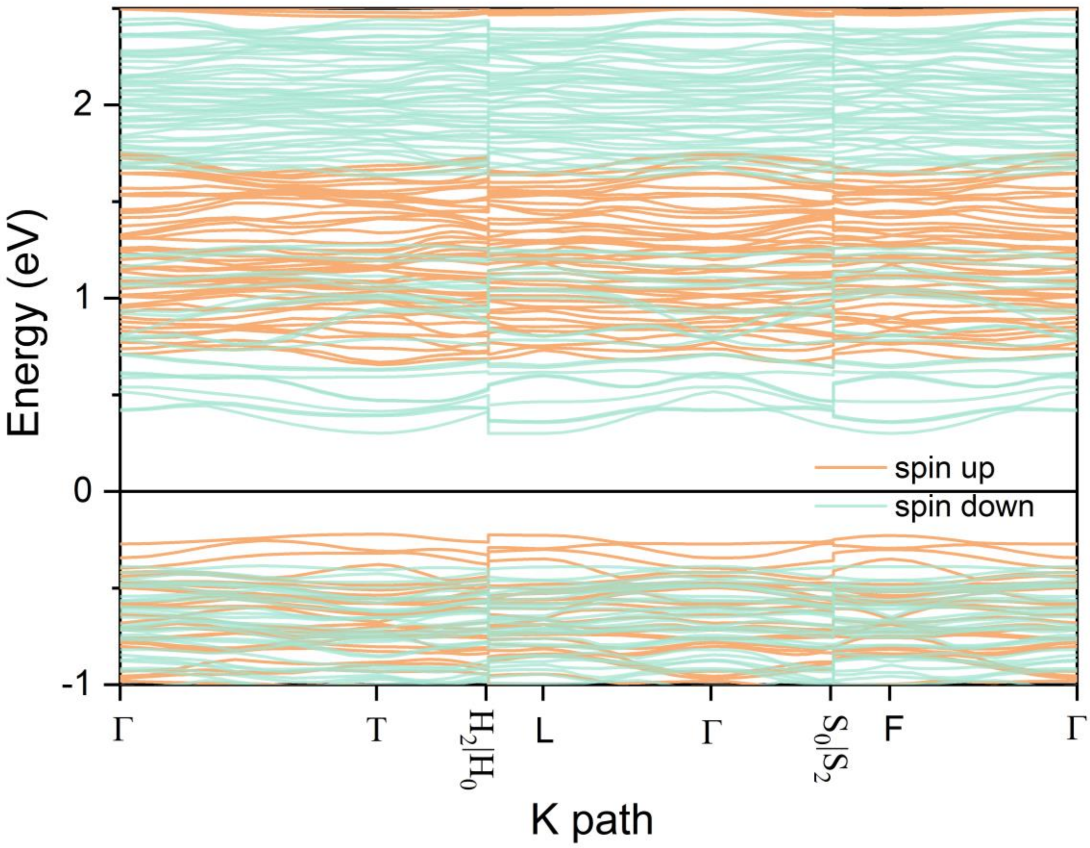

Supplementary Figure 18. Calculated band structure of $Li_{0.3}CoO_2$. This band structure is the calculated result from constructed supercell $Li_9Co_{30}O_{60}$ in Supplementary Figure 12.

Supplementary Tables

Supplementary Table 1. The *d*-spacing, corresponding CL aperture and convergent semi-angle used to collect CBED patterns for $LiCoO_2$.

hkl	*d*-spacing (Å)	Aperture diameter (μm)	Convergent semi-angle (mrad)
003	4.67367	20	2.19
$0\bar{1}1$	2.40000	50	5.11
006	2.33683	20	2.19
012	2.30101	50	5.11
$0\bar{1}4$	2.00035	50	5.11

Supplementary Table 2. The electron beam currents and dose rate of the two different experimental conditions.

CL aperture diameter (μm)	50	20
convergent semi-angle (mrad)	5.11	1.87
Spot diameter (nm)	0.98	2.19
Beam current (nA)	0.71	0.15
Dose rate ($e/s \cdot Å^2$)	5.87×10^7	2.44×10^5

Supplementary Table 3. Low-order structure factors measurement of $LiCoO_2$ using CBED.

Reflection	F_E^{CBED}	χ^2	F_X^{Con}	F_X^{Obs}
**003**	-12.0281	0.128	-75.2026	-74.7950
$\mathbf{0\bar{1}1}$	-7.7820	8.255	-54.8774	-54.9590
**006**	5.2031	0.128	35.0276	35.3030
**012**	4.6897	2.640	32.9610	33.7800
$\mathbf{0\bar{1}4}$	17.6667	5.131	88.8956	90.5540

F_E^{CBED} denotes the refined electron structure factors from CBED. χ^2 is the goodness of fit. F_X^{Con} represents the X-ray structure factors converted from electron-structure factors using the Mott-Bethe formula. F_X^{Obs} stands for the X-ray structure factors obtained using the synchrotron powder XRD.

Supplementary Table 4. Low-order structure factors measurement of $Li_{0.6}CoO_2$ using CBED.

Reflection	F_E^{CBED}	χ^2	F_X^{Con}	F_X^{Obs}
**003**	-15.2838	2.447	-80.4759	-80.0610
**0$\bar{1}$1**	-8.2820	1.235	-56.2763	-57.5079
**006**	4.0447	2.447	36.9176	35.1539
**012**	4.8031	4.154	29.4540	30.1469
**0$\bar{1}$4**	17.6636	2.345	84.4921	86.3889

F_E^{CBED} denotes the refined electron structure factors from CBED. χ^2 is the goodness of fit. F_X^{Con} represents the X-ray structure factors converted from electron-structure factors using the Mott-Bethe formula. F_X^{Obs} stands for the X-ray structure factors obtained using the synchrotron powder XRD.

Supplementary Table 5. Low-order structure factors measurement of $Li_{0.4}CoO_2$ using CBED.

Reflection	F_E^{CBED}	χ^2	F_X^{Con}	F_X^{Obs}
**003**	-19.0487	0.439	-82.2019	-81.8140
**$0\bar{1}1$**	-7.8576	0.483	-58.3635	-57.0550
**006**	4.8761	0.439	36.2501	37.2120
**012**	3.2848	2.107	30.9666	31.0820
**$0\bar{1}4$**	16.6159	0.976	84.6782	87.9260

F_E^{CBED} denotes the refined electron structure factors from CBED. χ^2 is the goodness of fit. F_X^{Con} represents the X-ray structure factors converted from electron-structure factors using the Mott-Bethe formula. F_X^{Obs} stands for the X-ray structure factors obtained using the synchrotron powder XRD.

Supplementary Table 6. Low-order structure factors measurement of $Li_{0.3}CoO_2$ using CBED.

Reflection	F_E^{CBED}	χ^2	F_X^{Con}	F_X^{Obs}
**003**	-17.0316	0.463	-83.3051	-82.5350
**$0\bar{1}1$**	-8.5585	1.364	-58.1658	-57.2490
**006**	3.6727	0.463	36.4529	35.9679
**012**	3.3336	0.414	29.9553	31.4750
**$0\bar{1}4$**	15.7307	1.372	86.4530	86.9929

F_E^{CBED} denotes the refined electron structure factors from CBED. χ^2 is the goodness of fit. F_X^{Con} represents the X-ray structure factors converted from electron-structure factors using the Mott-Bethe formula. F_X^{Obs} stands for the X-ray structure factors obtained using the synchrotron powder XRD.

Supplementary Table 7. Results from multipole refinement of the experimental data.

Parameters	$LiCoO_2$	$Li_{0.6}CoO_2$	$Li_{0.4}CoO_2$	$Li_{0.3}CoO_2$
$(sin\theta/\lambda)_{max}$ (Å^{-1})	1.2	1.2	1.2	1.2
$R(F^2)$ (%)	1.39	1.86	1.49	1.04
$wR(F^2)$ (%)	2.10	2.12	2.20	1.58
N_{refl}	222	217	219	220
U_{11}, U_{33}(Li) (Å^2)	0.006246 (1125)<br>0.010248 (1818)	0.012628 (2809)<br>0.005017 (2961)	0.00933 (3309)<br>0.004931 (3561)	0.007591 (2780)<br>0.004703 (3253)
U_{11}, U_{33}(Co) (Å^2)	0.001333 (172)<br>0.002367 (186)	0.001842 (204)<br>0.001596 (231)	0.000883 (191)<br>0.002034 (192)	0.001166 (156)<br>0.001885 (156)
U_{11}, U_{33}(O) (Å^2)	0.003567 (612)<br>0.000403 (1269)	0.002885 (275)<br>0.003163 (374)	0.000839 274)<br>0.008891 (385)	0.001729 (212)<br>0.006926 (248)
P_v(Co)	6.50(22)	6.44(27)	6.66 (33)	7.24 (29)
P_v(O)	6.75(11)	6.58 (13)	6.37(16)	6.03(14)
P_{20}, P_{40}, P_{43+}(Co)	0.291(57)<br>0.367(46)<br>-0.296(42)	0.103 (69)<br>0.228 (39)<br>-0.040 (32)	0.763 (216)<br>1.698 (371)<br>0.790 (364)	0.449 (273)<br>0.893 (502)<br>1.256 (423)
P_{10}, P_{20}, P_{30}, P_{33+}, P_{40}, P_{43+}(O)	-0.003(40)<br>0.295(97)<br>-0.032(24)<br>-0.003(25)<br>0.075(30)<br>-0.063(31)	-0.056 (83)<br>0.228 (103)<br>0.031 (95)<br>0.153 (98)<br>0.220 (104)<br>-0.139 (87)	0.016 (85)<br>0.117 (101)<br>0.064 (101)<br>0.001 (89)<br>0.173 (156)<br>0.120 (134)	-0.225 (123)<br>0.115 (132)<br>-0.094 (131)<br>0.267 (200)<br>0.514 (182)<br>0.014 (187)
κ, κ'(Co)	1.03(4), 1.95(9)	1.06(5), 2.20(25)	1.05(5), 0.62(4)	1.04(3), 0.55(5)
κ, κ'(O)	1.00(2), 2.12(4)	1.00 (2), 0.79 (14)	1.00(2), 0.82(24)	1.02(2), 0.56(10)

Supplementary Table 8. Lattice parameters, atomic coordinates, bond length, and bond angle of $Li_{1-x}CoO_2$.

Parameters	$LiCoO_2$	$Li_{0.6}CoO_2$	$Li_{0.4}CoO_2$	$Li_{0.3}CoO_2$
a (Å)	2.812792(4)	2.812155(14)	2.80962(4)	2.808697(16)
c (Å)	14.02096(3)	14.17254(18)	14.2662(6)	14.2924(2)
z(O)	0.23989(5)	0.23646(8)	0.23501(15)	0.23541(13)
Co-O bond length (Å)	1.9213(4)	1.9012(6)	1.8927(12)	1.8960(10)
O-O spacing (Å)	2.6179(12)	2.5592(18)	2.537(4)	2.548(3)
CoO_6 angle (°)	85.89(3)	84.61(4)	84.16(8)	84.42(7)

Supplementary Table 9. Calculated orbital populations of Co and O atoms in $Li_{1-x}CoO_2$ from DFT+*U*.

SOC	Co P_{3d}	Co t_{2g}	Co e_g	O $P_{valence}$
$LiCoO_2$	7.21	5.80	1.41	7.01
$Li_{0.625}CoO_2$	7.14	5.48	1.66	6.94
$Li_{0.4375}CoO_2$	7.1	5.32	1.78	6.90
$Li_{0.3125}CoO_2$	7.01	4.32	2.69	6.89

Supplementary Table 10. ***3d*****-orbital population of the Co atom in** $\mathbf{LiCoO_2}$**.**

	Co *3d*	Co t_{2g}	Co e_g
Experimental	6.50	4.83	1.67
Theoretical	6.72	5.04	1.68

Supplementary Table 11. Crystallographic data of constructed $Li_{30}Co_{30}O_{60}$ for pristine $LiCoO_2$.

Space group P1			
$a = 7.5078$ Å	$b = 9.8268$ Å $c = 14.1239$ Å	$\alpha = 90.0043°$ $\beta = 89.9978°$	$\gamma = 109.1068°$
Atom	x	y	z
Li1	0.0000	0.0000	0.0000
Li2	1.0000	0.5000	0.0000
Li3	0.2000	0.3000	0.0000
Li4	0.2000	0.8000	0.0000
Li5	0.8000	0.2000	0.0000
Li6	0.8000	0.7000	0.0000
Li7	0.6000	0.4000	0.0000
Li8	0.6000	0.9000	0.0000
Li9	0.4000	0.1000	0.0000
Li10	0.4000	0.6000	0.0000
Li11	0.2000	0.1333	0.3333
Li12	0.2000	0.6333	0.3333
Li13	0.4000	0.4333	0.3333
Li14	0.4000	0.9333	0.3333
Li15	1.0000	0.3333	0.3333
Li16	1.0000	0.8333	0.3333
Li17	0.8000	0.0333	0.3333
Li18	0.8000	0.5333	0.3333
Li19	0.6000	0.2333	0.3333
Li20	0.6000	0.7333	0.3333
Li21	1.0000	0.1667	0.6667
Li22	1.0000	0.6667	0.6667
Li23	0.2000	0.4667	0.6667
Li24	0.2000	0.9667	0.6667
Li25	0.8000	0.3667	0.6667
Li26	0.8000	0.8667	0.6667
Li27	0.6000	0.0667	0.6667
Li28	0.6000	0.5667	0.6667
Li29	0.4000	0.2667	0.6667
Li30	0.4000	0.7667	0.6667
Co1	0.0000	0.0000	0.5000
Co2	1.0000	0.5000	0.5000
Co3	0.2000	0.3000	0.5000
Co4	0.2000	0.8000	0.5000
Co5	0.8000	0.2000	0.5000
Co6	0.8000	0.7000	0.5000
Co7	0.6000	0.4000	0.5000
Co8	0.6000	0.9000	0.5000
Co9	0.4000	0.1000	0.5000
Co10	0.4000	0.6000	0.5000

Co11	0.2000	0.1333	0.8333
Co12	0.2000	0.6333	0.8333
Co13	0.4000	0.4333	0.8333
Co14	0.4000	0.9333	0.8333
Co15	1.0000	0.3333	0.8333
Co16	1.0000	0.8333	0.8333
Co17	0.8000	0.0333	0.8333
Co18	0.8000	0.5333	0.8333
Co19	0.6000	0.2333	0.8333
Co20	0.6000	0.7333	0.8333
Co21	1.0000	0.1667	0.1667
Co22	1.0000	0.6667	0.1667
Co23	0.2000	0.4667	0.1667
Co24	0.2000	0.9667	0.1667
Co25	0.8000	0.3667	0.1667
Co26	0.8000	0.8667	0.1667
Co27	0.6000	0.0667	0.1667
Co28	0.6000	0.5667	0.1667
Co29	0.4000	0.2667	0.1667
Co30	0.4000	0.7667	0.1667
O1	0.0000	0.0000	0.2398
O2	0.0000	0.5000	0.2398
O3	0.2000	0.3000	0.2398
O4	0.2000	0.8000	0.2398
O5	0.8000	0.2000	0.2398
O6	0.8000	0.7000	0.2398
O7	0.6000	0.4000	0.2398
O8	0.6000	0.9000	0.2398
O9	0.4000	0.1000	0.2398
O10	0.4000	0.6000	0.2398
O11	0.0000	0.0000	0.7602
O12	0.0000	0.5000	0.7602
O13	0.2000	0.3000	0.7602
O14	0.2000	0.8000	0.7602
O15	0.8000	0.2000	0.7602
O16	0.8000	0.7000	0.7602
O17	0.6000	0.4000	0.7602
O18	0.6000	0.9000	0.7602
O19	0.4000	0.1000	0.7602
O20	0.4000	0.6000	0.7602
O21	0.2000	0.1333	0.5731
O22	0.2000	0.6333	0.5731
O23	0.4000	0.4333	0.5731
O24	0.4000	0.9333	0.5731

O25	0.0000	0.3333	0.5731
O26	0.0000	0.8333	0.5731
O27	0.8000	0.0333	0.5731
O28	0.8000	0.5333	0.5731
O29	0.6000	0.2333	0.5731
O30	0.6000	0.7333	0.5731
O31	0.2000	0.1333	0.0936
O32	0.2000	0.6333	0.0936
O33	0.4000	0.4333	0.0936
O34	0.4000	0.9333	0.0936
O35	0.0000	0.3333	0.0936
O36	0.0000	0.8333	0.0936
O37	0.8000	0.0333	0.0936
O38	0.8000	0.5333	0.0936
O39	0.6000	0.2333	0.0936
O40	0.6000	0.7333	0.0936
O41	0.0000	0.1667	0.9064
O42	0.0000	0.6667	0.9064
O43	0.2000	0.4667	0.9064
O44	0.2000	0.9667	0.9064
O45	0.8000	0.3667	0.9064
O46	0.8000	0.8667	0.9064
O47	0.6000	0.0667	0.9064
O48	0.6000	0.5667	0.9064
O49	0.4000	0.2667	0.9064
O50	0.4000	0.7667	0.9064
O51	0.0000	0.1667	0.4269
O52	0.0000	0.6667	0.4269
O53	0.2000	0.4667	0.4269
O54	0.2000	0.9667	0.4269
O55	0.8000	0.3667	0.4269
O56	0.8000	0.8667	0.4269
O57	0.6000	0.0667	0.4269
O58	0.6000	0.5667	0.4269
O59	0.4000	0.2667	0.4269
O60	0.4000	0.7667	0.4269

Supplementary Table 12. Crystallographic data of constructed $Li_{18}Co_{30}O_{60}$ for $Li_{0.6}CoO_2$.

Space group P1			
$a = 7.4751$ Å $b = 9.7643$ Å $c = 14.4353$ Å $\alpha = 89.2489°$ $\beta = 89.7010°$ $\gamma = 108.9211°$			
Atom	x	y	z
Li1	0.0012	0.0061	-0.0003
Li2	0.0012	0.5061	-0.0003
Li3	0.8104	0.2052	-0.0009
Li4	0.8104	0.7052	-0.0009
Li5	0.3959	0.0933	0.0008
Li6	0.3958	0.5933	0.0007
Li7	0.1987	0.1269	0.3335
Li8	0.1987	0.6269	0.3335
Li9	0.3898	0.4279	0.3341
Li10	0.3898	0.9279	0.3341
Li11	0.8043	0.0396	0.3324
Li12	0.8043	0.5396	0.3324
Li13	0.0072	0.1651	0.6669
Li14	0.0072	0.6651	0.6669
Li15	0.1935	0.4680	0.6664
Li16	0.1935	0.9680	0.6664
Li17	0.6001	0.0666	0.6666
Li18	0.6001	0.5666	0.6665
Co1	0.0001	-0.0017	0.5007
Co2	0.0001	0.4983	0.5007
Co3	0.2003	0.2991	0.5005
Co4	0.2003	0.7991	0.5005
Co5	0.8016	0.2006	0.4975
Co6	0.8016	0.7006	0.4975
Co7	0.5992	0.3981	0.5027
Co8	0.5992	0.8981	0.5027
Co9	0.4007	0.1000	0.4994
Co10	0.4007	0.6000	0.4994
Co11	0.2000	0.1349	0.8327
Co12	0.2000	0.6349	0.8327
Co13	0.3986	0.4326	0.8356
Co14	0.3986	0.9326	0.8356
Co15	-0.0006	0.3340	0.8332
Co16	-0.0006	0.8340	0.8332
Co17	0.7992	0.0330	0.8338
Co18	0.7992	0.5330	0.8338
Co19	0.6007	0.2351	0.8306
Co20	0.6007	0.7351	0.8306
Co21	0.0011	0.1681	0.1657
Co22	0.0011	0.6681	0.1657

Co23	0.1989	0.4657	0.1676
Co24	0.1989	0.9657	0.1676
Co25	0.7992	0.3668	0.1686
Co26	0.7991	0.8668	0.1686
Co27	0.5999	0.0668	0.1666
Co28	0.5999	0.5668	0.1666
Co29	0.4008	0.2670	0.1647
Co30	0.4008	0.7670	0.1647
O1	-0.0054	0.0009	0.2365
O2	-0.0054	0.5009	0.2365
O3	0.2061	0.2960	0.2361
O4	0.2061	0.7960	0.2361
O5	0.7999	0.1988	0.2333
O6	0.7999	0.6988	0.2333
O7	0.5967	0.3994	0.2362
O8	0.5967	0.8994	0.2362
O9	0.3951	0.0978	0.2360
O10	0.3951	0.5978	0.2360
O11	0.0035	0.0053	0.7634
O12	0.0035	0.5053	0.7634
O13	0.2018	0.3066	0.7645
O14	0.2019	0.8066	0.7645
O15	0.7994	0.1994	0.7636
O16	0.7994	0.6994	0.7636
O17	0.5976	0.4041	0.7661
O18	0.5976	0.9041	0.7661
O19	0.4029	0.0974	0.7635
O20	0.4029	0.5974	0.7635
O21	0.1970	0.1279	0.5702
O22	0.1970	0.6279	0.5702
O23	0.4002	0.4338	0.5701
O24	0.4002	0.9338	0.5701
O25	0.9972	0.3266	0.5690
O26	-0.0028	0.8266	0.5690
O27	0.7973	0.0361	0.5696
O28	0.7973	0.5361	0.5697
O29	0.6024	0.2291	0.5669
O30	0.6024	0.7291	0.5669
O31	0.2057	0.1333	0.0967
O32	0.2057	0.6333	0.0967
O33	0.3999	0.4348	0.0998
O34	0.3999	0.9348	0.0998
O35	0.9938	0.3380	0.0973
O36	-0.0062	0.8380	0.0973

O37	0.8047	0.0356	0.0973
O38	0.8047	0.5356	0.0973
O39	0.6033	0.2345	0.0971
O40	0.6033	0.7345	0.0971
O41	0.9954	0.1628	0.9034
O42	0.9954	0.6628	0.9034
O43	0.1993	0.4681	0.9032
O44	0.1993	0.9681	0.9032
O45	0.7970	0.3617	0.9019
O46	0.7970	0.8617	0.9019
O47	0.5960	0.0701	0.9026
O48	0.5960	0.5701	0.9026
O49	0.4022	0.2639	0.8999
O50	0.4022	0.7639	0.8999
O51	0.0013	0.1651	0.4302
O52	0.0013	0.6651	0.4302
O53	0.2042	0.4700	0.4301
O54	0.2042	0.9700	0.4301
O55	0.7981	0.3694	0.4335
O56	0.7981	0.8694	0.4335
O57	0.6042	0.0628	0.4305
O58	0.6042	0.5628	0.4305
O59	0.4024	0.2716	0.4317
O60	0.4024	0.7716	0.4317

Supplementary Table 13. Crystallographic data of constructed $Li_{12}Co_{30}O_{60}$ for $Li_{0.4}CoO_2$.

Space group P1			
$a = 7.4667$ Å $b = 9.7499$ Å $c = 14.8052$ Å $\alpha = 88.5842°$ $\beta = 89.5399°$ $\gamma = 108.9251°$			
Atom	x	y	z
Li1	0.1828	0.2898	0.9991
Li2	0.1828	0.7898	0.9991
Li3	0.6408	0.4111	0.0007
Li4	0.6408	0.9111	0.0007
Li5	0.0171	0.3436	0.3349
Li6	0.0171	0.8436	0.3349
Li7	0.5618	0.2218	0.3323
Li8	0.5618	0.7218	0.3323
Li9	0.8198	0.3802	0.6662
Li10	0.8199	0.8802	0.6662
Li11	0.3828	0.2528	0.6665
Li12	0.3828	0.7527	0.6665
Co1	0.9992	0.0034	0.4988
Co2	0.9991	0.5034	0.4988
Co3	0.2000	0.3028	0.5003
Co4	0.2000	0.8028	0.5003
Co5	0.7995	0.2025	0.5022
Co6	0.7995	0.7025	0.5022
Co7	0.6004	0.4029	0.4979
Co8	0.6005	0.9029	0.4979
Co9	0.4009	0.1018	0.5012
Co10	0.4009	0.6018	0.5012
Co11	0.1992	0.1294	0.8341
Co12	0.1991	0.6294	0.8341
Co13	0.4044	0.4320	0.8308
Co14	0.4044	0.9321	0.8308
Co15	-0.0020	0.3305	0.8323
Co16	-0.0020	0.8305	0.8323
Co17	0.8029	0.0325	0.8312
Co18	0.8029	0.5324	0.8312
Co19	0.6028	0.2314	0.8349
Co20	0.6028	0.7314	0.8349
Co21	0.9965	0.1650	0.1688
Co22	0.9965	0.6650	0.1688
Co23	0.1996	0.4674	0.1658
Co24	0.1996	0.9675	0.1658
Co25	0.7984	0.3655	0.1665
Co26	0.7984	0.8655	0.1665
Co27	0.5981	0.0661	0.1671
Co28	0.5981	0.5661	0.1671

Co29	0.3980	0.2669	0.1680
Co30	0.3980	0.7669	0.1680
O1	0.9992	0.9940	0.2329
O2	0.9992	0.4940	0.2330
O3	0.1897	0.2991	0.2336
O4	0.1897	0.7991	0.2336
O5	0.7941	0.1965	0.2349
O6	0.7940	0.6965	0.2349
O7	0.5972	0.3959	0.2327
O8	0.5972	0.8959	0.2327
O9	0.3982	0.0970	0.2329
O10	0.3982	0.5969	0.2330
O11	0.0043	0.9992	0.7665
O12	0.0043	0.4991	0.7665
O13	0.2046	0.2971	0.7666
O14	0.2046	0.7970	0.7666
O15	0.8044	0.2064	0.7673
O16	0.8044	0.7064	0.7673
O17	0.6051	0.3990	0.7648
O18	0.6051	0.8991	0.7648
O19	0.4015	0.1043	0.7676
O20	0.4014	0.6043	0.7676
O21	0.1992	0.1353	0.5665
O22	0.1992	0.6353	0.5665
O23	0.3971	0.4295	0.5654
O24	0.3971	0.9295	0.5654
O25	0.9982	0.3371	0.5660
O26	0.9982	0.8371	0.5660
O27	0.8002	0.0279	0.5653
O28	0.8002	0.5279	0.5654
O29	0.5926	0.2338	0.5680
O30	0.5926	0.7338	0.5679
O31	0.1966	0.1386	0.1018
O32	0.1966	0.6386	0.1018
O33	0.4017	0.4355	0.0994
O34	0.4017	0.9356	0.0994
O35	0.0070	0.3336	0.1009
O36	0.0070	0.8336	0.1009
O37	0.7983	0.0353	0.1014
O38	0.7983	0.5353	0.1014
O39	0.5994	0.2364	0.1015
O40	0.5994	0.7364	0.1015
O41	0.0016	0.1635	0.8990
O42	0.0015	0.6635	0.8991

O43	0.2000	0.4578	0.8986
O44	0.2000	0.9579	0.8986
O45	0.8000	0.3643	0.8985
O46	0.7999	0.8644	0.8985
O47	0.5992	0.0559	0.8981
O48	0.5992	0.5559	0.8981
O49	0.3945	0.2635	0.9006
O50	0.3945	0.7636	0.9006
O51	0.0032	0.1765	0.4346
O52	0.0032	0.6765	0.4346
O53	0.2012	0.4700	0.4336
O54	0.2012	0.9700	0.4336
O55	0.8072	0.3715	0.4325
O56	0.8072	0.8715	0.4325
O57	0.6001	0.0777	0.4346
O58	0.6001	0.5777	0.4346
O59	0.4015	0.2683	0.4342
O60	0.4015	0.7684	0.4342

Supplementary Table 14. Crystallographic data of constructed $Li_9Co_{30}O_{60}$ for $Li_{0.3}CoO_2$.

Space group P1			
$a = 7.4786$ Å $b = 9.7698$ Å	$c = 14.7985$ Å	$\alpha = 90.9038°$ $\beta = 90.4686°$	$\gamma = 109.3570°$
Atom	x	y	z
Li1	0.1745	0.8174	0.0013
Li2	0.8000	0.2000	0.0000
Li3	0.4255	0.5826	-0.0013
Li4	0.1751	0.1503	0.3349
Li5	0.4246	0.9167	0.3320
Li6	0.7998	0.5331	0.3335
Li7	0.1754	0.4833	0.6680
Li8	0.8002	0.8669	0.6665
Li9	0.4249	0.2497	0.6651
Co1	0.0018	0.0017	0.5017
Co2	-0.0004	0.4988	0.4987
Co3	0.2003	0.3009	0.5000
Co4	0.1994	0.8004	0.5022
Co5	0.8000	0.2000	0.5000
Co6	0.8000	0.7000	0.5000
Co7	0.5982	0.3983	0.4983
Co8	0.6004	0.9012	0.5013
Co9	0.3997	0.0991	0.5000
Co10	0.4006	0.5996	0.4978
Co11	0.1992	0.1337	0.8352
Co12	0.2005	0.6343	0.8330
Co13	0.3999	0.4326	0.8333
Co14	0.4005	0.9331	0.8307
Co15	0.0020	0.3351	0.8351
Co16	-0.0006	0.8323	0.8316
Co17	0.7999	0.0334	0.8331
Co18	0.8002	0.5335	0.8331
Co19	0.6004	0.2346	0.8345
Co20	0.5982	0.7317	0.8313
Co21	-0.0004	0.1654	0.1655
Co22	0.0018	0.6683	0.1687
Co23	0.1995	0.4669	0.1693
Co24	0.2001	0.9674	0.1667
Co25	0.8001	0.3666	0.1669
Co26	0.7998	0.8665	0.1669
Co27	0.5980	0.0649	0.1649
Co28	0.6006	0.5677	0.1684
Co29	0.4008	0.2663	0.1648
Co30	0.3995	0.7657	0.1670
O1	0.0032	0.0079	0.2319

O2	0.9981	0.5006	0.2347
O3	0.2046	0.2989	0.2324
O4	0.2032	0.8033	0.2333
O5	0.8007	0.2008	0.2294
O6	0.8031	0.6998	0.2342
O7	0.6054	0.4021	0.2333
O8	0.5991	0.9050	0.2316
O9	0.4023	0.1045	0.2324
O10	0.3996	0.5988	0.2322
O11	-0.0054	-0.0021	0.7667
O12	0.0009	0.4950	0.7684
O13	0.1977	0.2955	0.7676
O14	0.2004	0.8012	0.7678
O15	0.7993	0.1992	0.7706
O16	0.7969	0.7002	0.7658
O17	0.5968	0.3921	0.7681
O18	0.6019	0.8994	0.7653
O19	0.3954	0.1011	0.7676
O20	0.3968	0.5967	0.7667
O21	0.2036	0.1366	0.5661
O22	0.2046	0.6325	0.5653
O23	0.4025	0.4380	0.5657
O24	0.3993	0.9323	0.5650
O25	0.0032	0.3413	0.5651
O26	0.9977	0.8340	0.5678
O27	0.8031	0.0334	0.5670
O28	0.8010	0.5342	0.5627
O29	0.5993	0.2382	0.5648
O30	0.6048	0.7355	0.5660
O31	0.2008	0.1347	0.1017
O32	0.1974	0.6284	0.1013
O33	0.3958	0.4340	0.1017
O34	0.3963	0.9300	0.1005
O35	0.9953	0.3312	0.1008
O36	0.0002	0.8280	0.1020
O37	0.7967	0.0333	0.0996
O38	0.7993	0.5323	0.1041
O39	0.6020	0.2329	0.0989
O40	0.5964	0.7252	0.1019
O41	-0.0020	0.1671	0.9011
O42	0.0036	0.6748	0.8981
O43	0.2037	0.4700	0.8995
O44	0.2042	0.9660	0.8983
O45	0.8033	0.3667	0.9004

O46	0.8007	0.8677	0.8959
O47	0.6047	0.0688	0.8992
O48	0.5998	0.5720	0.8980
O49	0.3992	0.2653	0.8983
O50	0.4026	0.7716	0.8987
O51	0.0007	0.1618	0.4352
O52	0.9952	0.6645	0.4340
O53	0.2007	0.4677	0.4350
O54	0.1975	0.9620	0.4343
O55	0.7969	0.3666	0.4330
O56	0.7990	0.8658	0.4373
O57	0.5968	0.0587	0.4349
O58	0.6023	0.5660	0.4322
O59	0.3964	0.2634	0.4339
O60	0.3954	0.7675	0.4347

Supplementary Table 15. Experimentally measured X-ray structure factors of $Li_{1-x}CoO_2$.

h	k	l	$LiCoO_2$	$Li_{0.6}CoO_2$	$Li_{0.4}CoO_2$	$Li_{0.3}CoO_2$
0	0	3	-75.20	-80.48	-82.20	-83.31
1	0	1	-54.88	-56.28	-58.36	-58.17
0	0	6	35.03	36.92	36.25	36.45
1	-1	2	32.96	29.45	30.97	29.96
1	0	4	88.89	84.49	84.68	86.45
1	-1	5	-42.10	-40.04	-40.21	-40.77
0	0	9	-33.50	-33.20	-34.93	-32.88
1	0	7	-54.87	-62.26	-60.14	-60.74
1	-1	8	68.03	68.37	62.26	63.34
2	-1	0	72.92	71.23	72.18	70.66
2	-1	3	-46.56	-47.29	-49.51	-49.90
1	0	10	31.19	34.37	34.84	34.80
2	-2	1	-38.78	-41.03	-40.60	-40.55
2	-1	6	29.73	28.68	30.40	29.68
2	0	2	29.13	27.62	26.12	26.57
0	0	12	54.20	51.54	48.14	49.84
2	-2	4	60.44	54.90	55.47	55.72
1	-1	11	-45.66	-51.13	-51.02	-51.72
2	0	5	-31.93	-30.40	-30.56	-30.97
2	-1	9	-26.20	-27.41	-25.23	-25.46
2	-2	7	-40.75	-42.74	-43.31	-43.47
2	0	8	48.51	46.79	46.56	45.17
1	0	13	-22.00	-21.78	-23.04	-22.58
0	0	15	-39.88	-43.52	-42.21	-43.13
1	-1	14	27.31	30.59	31.97	31.74
2	-2	10	25.99	26.22	28.37	27.68
3	-1	1	-30.62	-31.97	-32.57	-31.81
3	-2	2	23.94	22.55	21.66	21.15
2	-1	12	41.41	39.05	37.17	37.46
3	-1	4	45.82	43.35	41.75	42.05
2	0	11	-35.11	-38.24	-38.54	-39.13
3	-2	5	-26.25	-24.71	-25.82	-25.85
3	-1	7	-32.15	-34.14	-36.16	-35.45
1	0	16	35.60	32.75	30.20	30.80
3	-2	8	40.88	37.73	37.58	37.88
3	0	0	42.91	42.34	41.66	40.36
2	-2	13	-18.97	-19.21	-19.03	-19.28
3	-3	3	-29.67	-30.34	-31.19	-30.87
3	0	3	-29.67	-30.34	-31.19	-30.87
1	-1	17	-17.48	-17.12	-18.73	-18.17
0	0	18	23.76	29.06	30.14	28.83
2	-1	15	-33.16	-36.74	-35.34	-36.58

2	0	14	21.62	24.97	25.88	25.87
3	-1	10	20.25	20.76	22.32	21.86
3	-3	6	20.16	19.07	19.36	19.50
3	0	6	20.16	19.07	19.36	19.50
3	-2	11	-31.19	-32.54	-32.88	-33.21
3	0	9	-19.09	-18.44	-19.12	-18.74
3	-3	9	-19.09	-18.44	-19.12	-18.74
2	-2	16	31.04	28.49	27.53	26.89
1	0	19	-31.26	-33.98	-33.14	-32.79
4	-2	0	37.15	36.33	36.78	35.06
3	-1	13	-15.67	-16.59	-16.68	-16.54
4	-2	3	-26.35	-26.84	-27.38	-26.96
2	0	17	-14.92	-15.25	-15.90	-15.87
2	-1	18	21.29	25.36	25.23	25.13
3	-2	14	19.71	21.22	21.96	22.24
4	-3	1	-23.75	-23.44	-24.40	-23.68
1	-1	20	26.94	23.65	20.70	22.15
4	-2	6	17.38	17.43	17.49	16.92
4	-1	2	17.21	15.98	15.17	14.86
0	0	21	-14.89	-15.37	-17.27	-16.81
3	0	12	30.64	27.17	27.91	27.79
3	-3	12	30.64	27.17	27.91	27.79
4	-3	4	35.08	32.48	32.76	32.09
4	-1	5	-19.71	-18.81	-18.27	-18.40
4	-2	9	-17.17	-16.80	-16.37	-16.61
4	-3	7	-26.63	-26.95	-28.78	-27.97
3	-1	16	26.58	24.77	23.57	23.95
2	-2	19	-28.21	-30.10	-28.73	-29.41
4	-1	8	31.08	28.84	28.71	28.63
1	0	22	20.83	24.24	24.88	24.41
3	-2	17	-13.47	-13.53	-14.70	-14.54
3	0	15	-26.88	-29.25	-29.00	-29.34
3	-3	15	-26.88	-29.25	-29.00	-29.34
4	-3	10	17.27	16.94	17.63	17.54
4	0	1	-22.15	-20.60	-21.05	-21.15
2	0	20	23.63	21.72	19.48	20.27
4	-4	2	15.46	14.43	13.79	13.90
2	-1	21	-12.74	-13.71	-15.40	-15.12
4	-2	12	28.07	25.01	25.49	25.55
4	0	4	30.99	28.81	29.71	28.81
4	-1	11	-24.76	-26.64	-27.68	-27.11
4	-4	5	-18.79	-18.00	-16.81	-17.02
1	-1	23	-27.26	-27.72	-24.45	-25.73
0	0	24	20.18	18.65	16.71	16.89

4	0	7	-23.21	-24.53	-24.46	-24.19
3	-1	19	-25.78	-27.03	-24.98	-25.82
4	-4	8	30.17	26.87	26.70	27.13
4	-3	13	-13.99	-12.94	-13.41	-13.82
2	-2	22	18.69	22.61	22.55	23.26
3	0	18	17.20	20.00	21.69	21.02
3	-3	18	17.20	20.00	21.69	21.02
4	-2	15	-25.27	-26.23	-26.74	-27.00
4	-1	14	15.00	17.05	19.34	18.61
4	0	10	15.31	15.80	16.66	16.06
5	-2	1	-20.09	-19.82	-20.09	-19.61
3	-2	20	22.13	19.15	17.89	18.56
5	-3	2	14.34	13.87	13.31	12.78
5	-2	4	28.48	26.76	27.72	26.58
4	-4	11	-24.26	-24.98	-26.00	-25.04
5	-3	5	-17.45	-16.29	-16.18	-15.91
1	0	25	-10.92	-13.24	-15.65	-14.85
2	0	23	-23.93	-25.64	-23.03	15.57
2	-1	24	18.85	17.19	15.67	-23.67
5	-2	7	-22.33	-22.83	-25.41	-24.37
4	-3	16	23.11	20.83	20.39	20.55
5	-3	8	26.43	25.19	25.49	24.97
5	-1	0	28.44	26.99	27.59	26.46
4	0	13	-12.02	-12.49	-12.61	-12.69
5	-4	3	-20.88	-20.51	-21.23	-20.82
5	-1	3	-20.88	-20.51	-21.23	-20.82
1	-1	26	19.20	23.96	22.63	23.43
3	-1	22	16.84	21.51	21.60	21.24
4	-1	17	-11.40	-11.27	-12.24	-12.13
4	-2	18	15.89	18.46	19.71	19.12
4	-4	14	15.36	15.78	17.64	16.89
0	0	27	-22.87	-23.71	-19.79	-21.16
5	-2	10	13.84	14.19	15.22	14.85
5	-1	6	13.29	12.89	13.05	13.02
5	-4	6	13.29	12.89	13.05	13.02
3	0	21	-10.49	-11.78	-13.28	-12.79
3	-3	21	-10.49	-11.78	-13.28	-12.79
5	-3	11	-22.97	-22.80	-25.26	-24.19
2	-2	25	-10.92	-12.25	-14.86	-13.85
3	-2	23	-23.60	-23.57	-21.82	-22.57
5	-1	9	-14.31	-13.48	-13.26	-13.18
5	-4	9	-14.31	-13.48	-13.26	-13.18
4	0	16	20.56	19.20	19.15	18.86
4	-3	19	-23.39	-23.59	-23.95	-23.50

5	-2	13	-12.08	-11.58	-12.05	-12.10
2	0	26	17.99	21.99	21.60	21.24
1	0	28	16.09	13.86	14.10	13.56
4	-4	17	-11.84	-10.81	-11.61	-11.57
5	-3	14	14.87	15.11	16.53	16.24
2	-1	27	-22.69	-21.88	-18.52	-20.30
5	-5	1	-17.88	-17.29	-16.82	-16.92
4	-1	20	19.07	16.94	15.64	16.27
5	0	2	12.61	10.88	10.92	11.06
4	-2	21	-10.19	-11.03	-12.48	-11.99
5	-1	12	22.99	20.07	20.36	20.46
5	-4	12	22.99	20.07	20.36	20.46
5	-5	4	25.75	24.75	24.05	23.17
5	0	5	-15.49	-14.08	-13.82	-13.64
3	-1	25	-9.62	-11.65	-13.79	-13.09
1	-1	29	-9.44	-13.79	-14.93	-14.23
3	-3	24	16.83	14.44	13.65	13.65
3	0	24	16.83	14.44	13.65	13.65
5	-5	7	-20.46	-19.91	-22.15	-21.05
5	-2	16	19.99	18.00	18.07	17.90
4	0	19	-21.09	-21.98	-22.53	-22.54
5	0	8	23.14	21.90	22.58	22.06
6	-3	0	25.01	23.81	24.10	23.44
0	0	30	18.22	22.04	19.99	20.05
6	-3	3	-19.06	-18.14	-20.09	-18.83
3	-2	26	17.01	20.84	20.17	20.01
4	-3	22	16.04	19.19	19.54	18.99
2	-2	28	15.27	13.14	13.20	12.92
5	-3	17	-11.40	-10.10	-11.52	-11.17
5	-1	15	-21.54	-21.61	-23.25	-22.44
5	-4	15	-21.54	-21.61	-23.25	-22.44
5	-5	10	12.71	13.39	14.39	13.65
6	-4	1	-17.00	-16.84	-17.48	-16.60
4	-4	20	19.00	15.90	15.46	15.53
6	-3	6	12.19	12.02	12.28	11.88
6	-2	2	12.08	11.28	11.01	10.74
6	-4	4	24.15	22.03	23.40	23.02
5	0	11	-19.75	-20.71	-22.21	-21.64
6	-2	5	-14.85	-13.36	-13.89	-13.95
4	-1	23	-20.58	-21.46	-19.86	-20.42
2	0	29	-9.12	-12.63	-14.65	-13.92
4	-2	24	16.00	13.81	13.21	13.45
6	-3	9	-13.45	-18.73	-15.61	-16.85
6	-4	7	-19.56	-19.46	-20.96	-20.16

5	-2	19	-21.08	-21.14	-20.97	-21.17
6	-2	8	22.61	21.71	21.45	20.75
1	0	31	-20.53	-18.73	-15.61	-16.85
5	-5	13	-12.89	-10.96	-10.80	-10.87
2	-1	30	17.39	21.25	18.78	19.42
4	0	22	15.04	17.45	18.82	18.06
3	-1	28	14.39	12.54	12.43	12.20
5	-1	18	13.62	15.32	17.84	16.79
5	-4	18	13.62	15.32	17.84	16.79
5	0	14	12.93	13.91	15.85	15.07
3	0	27	-20.48	-19.56	-17.46	-18.15
3	-3	27	-20.48	-19.56	-17.46	-18.15
6	-4	10	12.24	12.89	13.63	13.18
6	-1	1	-16.35	-16.32	-16.55	-16.17
5	-3	20	18.31	15.59	14.83	15.12
6	-5	2	11.73	10.32	10.36	10.51
6	-3	12	20.34	18.59	19.37	19.16
6	-1	4	22.40	20.68	22.97	21.82
6	-2	11	-18.98	-19.65	-21.63	-20.56
6	-5	5	-14.81	-12.93	-12.87	-12.80
4	-3	25	-9.43	-10.71	-13.02	-12.23
1	-1	32	12.43	11.30	12.65	11.96
4	-4	23	-20.65	-12.93	-19.17	-19.55
3	-2	29	-8.63	-12.18	-14.16	-13.37
6	-1	7	-17.80	-18.18	-19.67	-19.34
5	-5	16	18.46	16.72	16.05	16.35
0	0	33	-8.15	-13.97	-14.91	-14.42
6	-5	8	21.63	20.00	21.19	20.03
2	-2	31	-19.34	-17.86	-15.02	-15.95
6	-4	13	-11.42	-10.34	-10.62	-10.56
4	-1	26	14.54	19.05	18.40	18.19
5	-2	22	13.47	17.23	18.42	17.41
5	0	17	-9.79	-9.45	-10.56	-10.38
6	-3	15	-19.06	-19.92	-21.60	-20.87
6	-2	14	12.52	13.06	14.87	14.03
4	-2	27	-18.59	-19.02	-16.91	-17.49
6	-1	10	11.19	11.31	12.23	12.00
5	-1	21	-9.00	-9.62	-11.28	-10.70
5	-4	21	-9.00	-9.62	-11.28	-10.70
6	-5	11	-19.18	-18.71	-21.34	-20.26
4	0	25	-9.22	-10.34	-12.39	-11.74
2	0	32	11.36	10.64	11.90	11.23
5	-3	23	-19.49	-19.39	-18.54	-18.76
6	-4	16	18.43		16.72	-20.26

2 -1 33	-9.49		-14.34	-13.91
1 0 34	17.12			17.55
5 -5 19	-21.31			
6 0 0	22.07			

Supplementary References

1. Ma C, *et al.* Strong Coupling of the Iron-Quadrupole and Anion-Dipole Polarizations in Ba(Fe1-xCox)(2)As-2. *Physical Review Letters* **112**, (2014).
2. Zuo JM, Weickenmeier AL. ON THE BEAM SELECTION AND CONVERGENCE IN THE BLOCH-WAVE METHOD. *Ultramicroscopy* **57**, 375-383 (1995).
3. Václav P, Michal D, Lukáš P. Crystallographic Computing System JANA2006: General features. *Zeitschrift für Kristallographie - Crystalline Materials* **229**, 345-352 (2014).
4. Hansen NK, Coppens P. Testing aspherical atom refinements on small-molecule data sets. *Acta Crystallographica Section A* **34**, 909-921 (1978).
5. Stevens ED, Coppens P. Refinement of metal d-orbital occupancies from X-ray diffraction data. *Acta Crystallographica Section A* **35**, 536-539 (1979).
6. Spence JCH, Zuo JM. Electron Microdiffraction Ch. 4 (Springer Science+Business Media New York 1992).
7. Blaha; P*, et al. WIEN2K: An Augmented Plane Wave Plus Local Orbitals Program for Calculating Crystal Properties* (2021).
8. Blaha P, Schwarz K, Sorantin P, Trickey SB. Full-potential, linearized augmented plane wave programs for crystalline systems. *Computer Physics Communications* **59**, 399-415 (1990).
9. Kresse G, Furthmüller J. Efficient iterative schemes for ab initio total-energy calculations using a plane-wave basis set. *Physical Review B* **54**, 11169-11186 (1996).
10. Kresse G, Furthmüller J. Efficiency of ab-initio total energy calculations for metals and semiconductors using a plane-wave basis set. *Computational Materials Science* **6**, 15-50 (1996).
11. Blöchl PE. Projector augmented-wave method. *Physical Review B* **50**, 17953-17979 (1994).
